# Sequencing introduced false positive rare taxa lead to biased microbial community diversity, assembly, and interaction interpretation in amplicon studies

**DOI:** 10.1186/s40793-022-00436-y

**Published:** 2022-08-17

**Authors:** Yangyang Jia, Shengguo Zhao, Wenjie Guo, Ling Peng, Fang Zhao, Lushan Wang, Guangyi Fan, Yuanfang Zhu, Dayou Xu, Guilin Liu, Ruoqing Wang, Xiaodong Fang, He Zhang, Karsten Kristiansen, Wenwei Zhang, Jianwei Chen

**Affiliations:** 1 BGI-Shenzhen, Shenzhen, 518083 China; 2 BGI-Qingdao, BGI-Shenzhen, Qingdao, 266555 China; 3 State Key Laboratory of Animal Nutrition, Institute of Animal Sciences, Chinese Academy of Agricultural Sciences, Beijing, 100193 China; 4 State Key Laboratory of Microbial Technology, Institute of Microbial Technology, Shandong University, Qingdao, 266237 China; 5 Department of Biology, Hong Kong Baptist University, Hong Kong, China; 6 Laboratory of Genomics and Molecular Biomedicine, Department of Biology, University of Copenhagen, Universitetsparken 13, 2100 Copenhagen, Denmark; 7 Qingdao-Europe Advanced Institute for Life Sciences, BGI-Shenzhen, Qingdao, 266555 China

**Keywords:** Amplicon sequencing, Microbial rare taxa, Index misassignment, Community assembly, Keystone species

## Abstract

**Background:**

Increasing studies have demonstrated potential disproportionate functional and ecological contributions of rare taxa in a microbial community. However, the study of the microbial rare biosphere is hampered by their inherent scarcity and the deficiency of currently available techniques. Sample-wise cross contaminations might be introduced by sample index misassignment in the most widely used metabarcoding amplicon sequencing approach. Although downstream bioinformatic quality control and clustering or denoising algorithms could remove sequencing errors and non-biological artifact reads, no algorithm could eliminate high quality reads from sample-wise cross contaminations introduced by index misassignment, making it difficult to distinguish between *bona fide* rare taxa and potential false positives in metabarcoding studies.

**Results:**

We thoroughly evaluated the rate of index misassignment of the widely used NovaSeq 6000 and DNBSEQ-G400 sequencing platforms using both commercial and customized mock communities, and observed significant lower (0.08% vs. 5.68%) fraction of potential false positive reads for DNBSEQ-G400 as compared to NovaSeq 6000. Significant batch effects could be caused by stochastically introduced false positive or false negative rare taxa. These false detections could also lead to inflated alpha diversity of relatively simple microbial communities and underestimated that of complex ones. Further test using a set of cow rumen samples reported differential rare taxa by different sequencing platforms. Correlation analysis of the rare taxa detected by each sequencing platform demonstrated that the rare taxa identified by DNBSEQ-G400 platform had a much higher possibility to be correlated with the physiochemical properties of rumen fluid as compared to NovaSeq 6000 platform. Community assembly mechanism and microbial network correlation analysis indicated that false positive or negative rare taxa detection could lead to biased community assembly mechanism and identification of fake keystone species of the community.

**Conclusions:**

We highly suggest proper positive/negative/blank controls, technical replicate settings, and proper sequencing platform selection in future amplicon studies, especially when the microbial rare biosphere would be focused.

**Supplementary Information:**

The online version contains supplementary material available at 10.1186/s40793-022-00436-y.

## Introduction

Microbial communities in various environments are usually composed of a skewed abundance of microbes with a few highly dominant taxa and numerous rare taxa as revealed by the “long tail” of the rank-abundance curve [1, 2]. The rare taxa, also known as the microbial “rare biosphere” [3], although exist in very low relative abundances, play important ecological roles in microbial communities. One of the most important roles is as the “seed bank” or the “hidden backbone” in maintaining the stability and robustness of microbial communities [4]. For example, rare taxa were claimed to be the major driver soil multifunctionality and played over-proportional role in biogeochemical cycles [5]. Some rare taxa play crucial ecological functions in various biogeochemical processes and in human health [4]. For example, Pester and colleagues demonstrated that *Desulfosporosinus*, despite detected with a relative abundance of less than 0.006%, had a fundamental role in sulfate reduction in a peatland ecosystem [6], and maintained high cellular activities under in situ-like conditions in lab [7]. Bodelier and colleagues found that the rare methane-oxidizing bacteria play an unneglectable role in the dynamics and consumption of methane in a wetland [8]. Some rare taxa were also found to be keystone species in a changing aquatic ecosystems by correlation network analysis [9], and others showed disproportionate high metabolic activities compared to their low relatively abundances in various environments, such as ocean [10], and anaerobic digesters [11], even air [12].

Although rising interests of the microbial rare taxa have been observed in recent years, our knowledge of the rare fraction of microbial communities is still in its infancy. High throughput sequencing, including shotgun metagenome sequencing of the entire DNA material of a community, and metabarcoding amplicon sequencing, normally targeting one or several of the highly variable region(s) of the small subunit ribosomal ribonucleic acid (SSU rRNA) [13, 14], represent the most widely used approaches to query microbial rare biosphere. However, sequencing errors may happen and contaminations might be introduced during the sequencing process. Although the low frequent errors and contaminations would not threaten study of the abundant microbial community, they greatly hampered the study of the rare fraction of the microbial community. One of the most challenging parts in studying the rare biosphere is to distinguish between sequences from the *bona fide* rare taxa and sequence artefacts introduced by PCR or sequencing error, and potential false positives represented by biological reads from various contaminations.

As reviewed by Lynch and Neufeld [13], sequencing errors introduced by low quality or ambiguous bases could normally be removed by setting stringent quality filtering threshold and clustering sequencing reads into Operational Taxonomic Units (OTUs) with certain sequence identity [15]. Post-clustering curation algorithm was also developed to remove sequencing introduced errors [16]. Chimeric sequences generated during PCR could normally be removed by bioinformatic algorithms [17]. Routinely used denoising algorithms, including DADA2 [18], Deblur [19] and Unoise3 [20], facilitated the analysis of amplicon sequences at the exact sequence variant level [21], revealing the microbial community compositions with finer resolution, and could eliminate part of the sequencing errors during clustering.

Despite the developments of algorithms and analyzing methods, all the high-throughput amplicon sequencing data filtering efforts made previously were focused on removing “artefacts”, that is non-biological sequences generated during the experimental process. None of the algorithms could remove potential sample-wise cross contaminations caused by index misassignment (also called index hopping) among samples pooled and sequenced in the same run, as they are high quality reads, not errors [22, 23]. Index misassignment could occur at a rate of 0.2 ~ 6% or even higher on various Illumina sequencing platforms [24], causing potential misinterpretation of the sequencing results. These negative consequences could be disastrous, especially for clinical diagnoses depending heavily on scarce mutations and/or rare microbes [23, 25–27]. Platforms using different sequencing technologies could have different *pros* and *cons*. Frequency of index misassignment of the DNBSEQ platform, which used a combinatorial Probe-Anchor Synthesis method and DNA nanoball sequencing technology developed by MGI, was demonstrated to be as low as 0.0001–0.0004% [28]. Studies evaluating these different sequencing platforms in whole genome sequencing have been conducted by researchers worldwide [29, 30]. In metagenomic studies, index misassignment has also been demonstrated to be an overlooked source of error in metabarcoding amplicon studies using pyrosequencing [22] or Illumina sequencing technology [31, 32] for a decade. However, there are currently still very few studies evaluating how and to what extend could index misassignment affect the study and understanding regarding the rare fraction of microbial communities systematically [33, 34].

To address these questions, in the present study, commercial and customized mock communities, and microbial communities with differential complexity from several typical ecosystems were sequenced at two different mainstream sequencing platforms to show how index misassignment could interfere the interpretation and understanding of the rare taxa in various microbial communities. In addition, a real case study of cow rumen microbial communities further demonstrated that index-misassignment could lead to biased microbial compositions, community assembly, and ecological roles of the microbial rare biosphere. Finally, best practices for both experimental setting and data processing were suggested to eliminate potential false positives introduced by index misassignment in amplicon studies.

## Results

### Less batch effects and fewer false positives on DNBSEQ-G400 platform

**Fig. 1:**
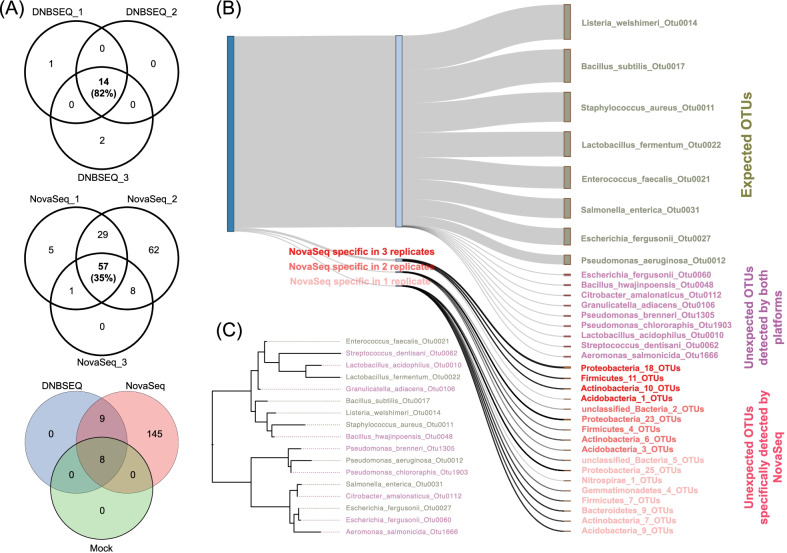
Amplicon sequencing of the commercial mock community. **A** The number of shared OTUs among technical triplicates for both DNBSEQ and Novaseq platforms, and the number of shared unique OTUs detected by pooled technical triplicates by different sequencing platforms and the theoretical members of the mock community. **B** Sankey diagram showing the phylogeny and number of unique OTUs detected by each sequencing platform. For the expected OTUs, the height of the bars was proportional to the averaged relative abundance reported by two sequencing platforms. OTUs detected by both DNBSEQ and NovaSeq, or specifically detected by NovaSeq platform were indicated and color coded. **C** Phylogenetic tree of the expected members of the microbes and OTUs detected by both sequencing platforms

In order to evaluate the frequency of potential false positives that might be introduced by index misassignment in amplicon sequencing, commercial mock community ZymoBIOMICS™ Microbial Community DNA Standard with known composition (Additional file 1: Table S1) and two customized mock communities, with one of which containing 4 known bacteria strains (4Bac), and the other containing 7 (7Bac), (Additional file 1: Table S2, Table S3) were subjected to amplicon sequencing of the 16S rRNA gene V4 region using both Illumina NovaSeq 6000 and MGI DNBSEQ-G400 platforms (Additional file 2: Fig. S1). For the commercial mock community, a total of 17 (14, 15, 16 for each replicate respectively) unique OTUs were obtained by DNBSEQ-G400 platform, with 3 of them observed once and the other 14 OTUs consistently observed by all three technical replicates. In comparison, a total of 162 (92, 156, 66 for each replicate respectively) unique OTUs were obtained by Illumina NovaSeq 6000 platform, with 67 OTUs observed once, 95 OTUs twice and 57 OTUs observed by all three technical replicates. Significant batch effect was observed for NovaSeq platform with only 35% of the OTUs being consistently observed by all three replicates compared to 82% of DNBSEQ (Fig. 1A). Comparison of the two customized mock communities revealed similar observations, with eight of eleven OTUs by DNBSEQ while 39 of 85 OTUs by NovaSeq platform for the 4Bac community; and nine of eleven by DNBSEQ while 42 of 83 OTUs by NovaSeq platform for the 7Bac community, consistently detected by all three technical replicates (Additional file 3: Fig. S2A, B, Additional file 1: Tables S2 and S3).

Taxonomic annotation of the OTUs revealed that all members of the mock community were consistently detected by all the technical replicates on both sequencing platforms, indicating successful detection of abundant microbial members in a community given enough sequencing depth. Despite successful detection of the expected members by both sequencing platforms, both platforms reported certain amount of unexpected OTUs (i.e., OTUs could not find a match from the theoretical composition of the mock community), representing potential false positives. The number of unexpected OTUs for NovaSeq platform was almost two orders of magnitude higher than that of DNBSEQ platform. Relative abundances of the unexpected OTUs were up to 1.19% and 0.09%, accounting for a total of 5.68% and 0.08% reads for NovaSeq and DNBSEQ platform, respectively, for the commercial mock community (Fig. 1B, Additional file 1: Table S1). Similar trend was observed for both customized mock communities (Additional file 1: Tables S2 and S3).

### False positives might be introduced by index misassignment and could not be removed by routine QC process

Comparison of unexpected OTUs detected by each of the platforms showed that all OTUs observed by DNBSEQ platform were consistently observed by NovaSeq platform, indicating that these unexpected OTUs were more likely from the original sample, instead of from the respective sequencing process (Fig. 1B). Sequence alignment of the nine unexpected OTUs detected by both platforms indicated that five of them (Otu1903, Otu0048, Otu0106, Otu0112 and Otu0060) having a sequence identity of 97.18% ~ 99.60% to the mock bacteria. These OTUs might be different strains of their mock members. The other four OTUs (Otu0010, Otu0062, Otu1666 and Otu1305) also had 91.70% ~ 96.39% sequence identity to their mock members, but might not come from the theoretical mock community (Fig. 1C, Additional file 1: Table S1). Those OTUs from the same species of the mock members might be from single nucleotide variations (SNVs) of the original mock bacteria, while there was a chance for the OTUs with relatively low sequence identity to mock bacteria to be potential contaminants from the original DNA sample or contaminations from environment during aliquoting. On the other hand, taxonomic annotation of the unexpected OTUs specifically detected by NovaSeq platform revealed a highly diverse spectrum of phylogeny, with small shared fractions among technical replicates (Fig. 1B, Additional file 3:Fig. S2C). Amplicon sequencing of two customized mock communities consistently revealed more unexpected OTUs with diverse phylogeny detected by NovaSeq 6000 platform (Additional file 3: Fig. S2D, E, Additional files 1: Tables S2 and S3). Furthermore, comparison of distinct samples sequenced in the same batch revealed a considerable fraction of shared OTUs among samples on NovaSeq platform, including 37, 47 and 37 respectively (Additional file 4: Fig. S3).

In order to evaluate whether those potential contaminant reads could be removed in silico during data analysis, more stringent quality control process was used to filter the data before downstream clustering and statistical analysis. As index misassignment was supposed to happen at relatively low rate [23, 25–27], higher threshold of minimum tags to be contained in an OTU was set tring to remove potential low-rate false positives. However, a raised threshold of even 50 could not remove all the potential contaminants (Additional file 1: Tables S1, S2 and S3).

### The rare taxa sub-community were more vulnerable to biases

As real microbial communities occupying various ecosystems are much more complex than mock communities regarding both microbial composition and abundance distribution, we speculated that index misassignment might lead to more intriguing false positive and/or negative detections in real samples. Biological triplicate samples from several typical microbial ecosystems, including mice gut, surface seawater and mangrove sediment representing relatively low, moderate, and high diversity communities, were sequenced with technical triplicate on both DNBSEQ-G400 and NovaSeq 6000 platforms at comparable sequencing depth of around 60,000 reads. The number and fraction of clean reads after QC and number of obtained OTUs for each sample were summarized in Additional file 1: Table S4.

**Fig. 2:**
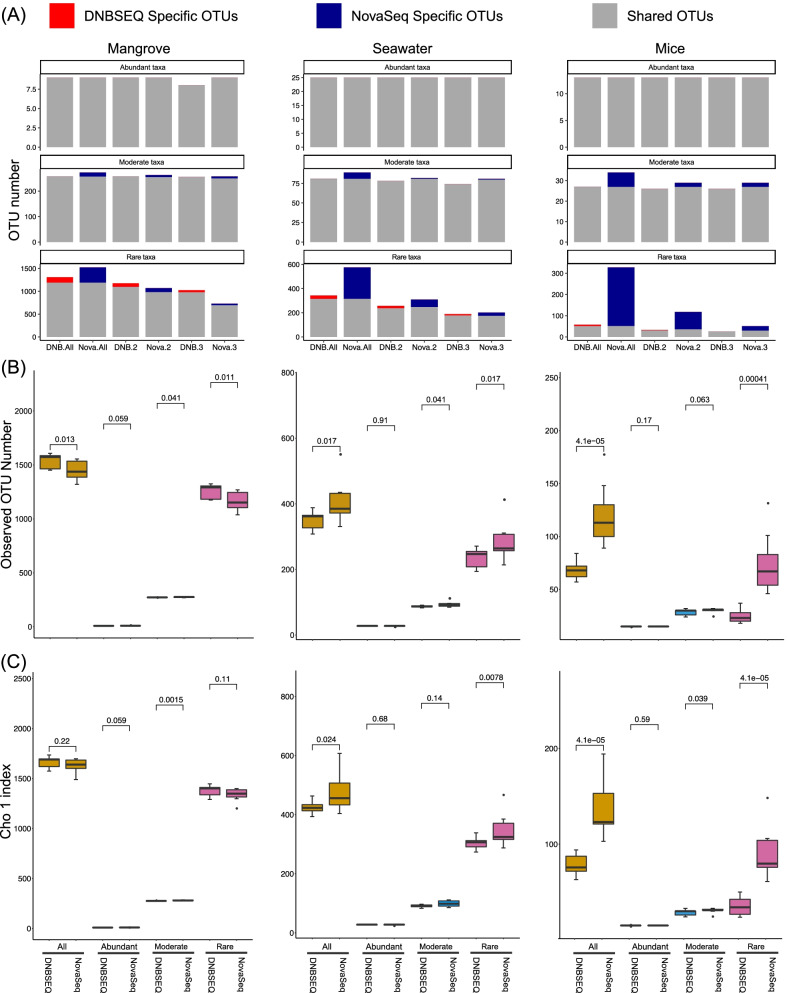
Comparison of the amplicon sequencing results of three typical ecological systems between DNBSEQ and NovaSeq sequencing platforms. **A** Evaluation of the reproducibility of the amplicon sequencing results of DNBSEQ and NovaSeq sequencing platform in revealing the membership of microbes of abundant, moderate and rate taxa subcommunity from ecosystems with various complexity (DNB All, DNB 2, DNB 3 denotes number of all unique OTUs detected by DNBSEQ platform, consistently detected by at least 2 technical replicates, and consistently detected by all three technical replicates, respectively. Similar naming scheme was used for the NovaSeq platform). **B**/**C** Comparison of the alpha diversity of the overall community, abundant sub-community, moderate sub-community, and rare sub-community as revealed by DNBSEQ and NovaSeq sequencing platforms based on Observed OTU Number **B** and Chao I index **C**

For these real samples from different microbial ecosystems, we hypothesized that the recovery rate of a taxon correlates strongly with its abundance. That is, abundant taxa have a much higher chance of being captured than the rare ones. Here we define taxa with a relative abundance of ≥ 1% as abundant, < 0.1% as rare, and the rest in between as moderate. Comparison of technical triplicates for each ecosystem revealed an average of 100% of abundant, 97.53% moderate and 68.93% rare taxa being consistently detected by more than one technical replicate by DNBSEQ-G400, while these fractions were 100%, 87.94% and 39.50% for NovaSeq platform (Fig. 2A, Additional file 5: Fig. S4A). Comparison between sequencing platforms revealed higher fractions of sequencing platform specific taxa of the moderate and rare sub-communities, especially for NovaSeq platform. The NovaSeq 6000 platform yielded significantly higher alpha diversity for the seawater and mice gut samples but significantly lower for the mangrove ones, as compared to the results of DNBSEQ-G400 platform (Fig. 2B, C).

In order to further evaluate whether those platform specific OTUs were more likely false positives or false negatives missed by the other platform, we mapped the shotgun metagenomic reads of the same samples to the OTU representative sequences of the mangrove (Additional file 5: Fig. S4B) [35] and mice gut (Additional file 5: Fig. S4C) (not published data) ecosystems. The NovaSeq platform yielded much more OTUs undetectable in the metagenomes than DNBSEQ-G400, indicating a higher potential risk for false positives. Furthermore, both weighted and unweighted UniFrac trees consistently showed that technical triplicates of the DNBSEQ-G400 results were grouped by their biological samples for all the tested ecosystems, while technical triplicates of more samples were grouped by sequencing batch or in a random way for NovaSeq sequencing results (Additional file 6: Fig. S5).

### The rumen microbial community revealed by different sequencing platforms

Cow rumen ecosystem not only harbors a diverse microbes capable of digesting insoluble lignocellulosic biomass into accessible carbon and energy sources for their host, but also have complex connections with many of the host attributes and performances [36–38]. A lot of researches have been done trying to elucidate the microbial compositional and functional diversity, but most of the previous studies ignored the rare taxa of the microbial populations [33, 34]. In order to study the ecological properties of rumen rare taxa, and evaluate the potential differences of the rare community revealed by different sequencing platforms, we sequenced a total of 47 cow rumen fluid samples using amplicon sequencing technology at both platforms with identical sequencing depth.

**Fig. 3:**
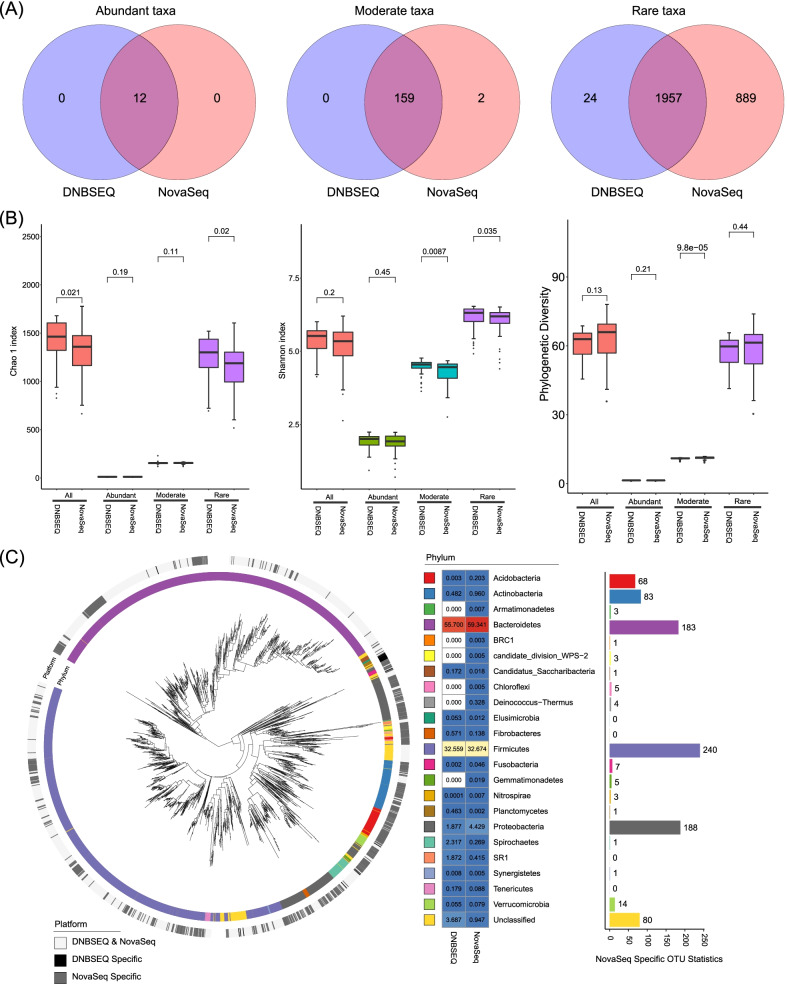
Characteristics of cow rumen microbial communities revealed by different sequencing platforms **A** Number of shared abundant, moderate, and rare unique OTUs detected by different sequencing platforms in cow rumen. **B** Comparison of Chao I index, Shannon index, and the phylogenetic diversity of the overall community, abundant sub-community, moderate sub-community, and rare sub-community as revealed by DNBSEQ and NovaSeq sequencing platforms in cow rumen. **C** Phylogenetic distribution of OTUs detected by both or either of the DNBSEQ or NovaSeq sequencing platforms in cow rumen. The inner circle was color coded by phylogeny, and the outer circle was color coded according to whether the OTU was consistently detected by both sequencing platforms, or specifically detected by either of DNBSEQ or NovaSeq sequencing platform. Heatmap in the middle panel shows the relative abundance of different phylum revealed by DNBSEQ or NovaSeq. Bar chart on the right panel shows the number of unique OTUs specifically detected by NovaSeq platform in different phylum

Of the 3043 OTUs clustered, only twelve were identified as abundant taxa, while 161 and 2870 taxa were identified as moderate and rare respectively. Almost identical abundant and moderate microbial taxa were revealed by DNBSEQ and NovaSeq, indicating relatively low sequencing platform effects with regard to the membership of these two sub-community (Fig. 3A). However, significant more sequencing platform specific taxa were observed for the rare microbial population, particulary for the NovaSeq sequencing platform. Of the 2870 rare OTUs, 913 (32%) were detected by only one platform. NovaSeq account for a large proportion of the platform specific OTUs (889 out of 913), much higher than DNBSEQ (only 24), and showed a much more diverse metacommunity (Fig. 3A).

**Fig. 4:**
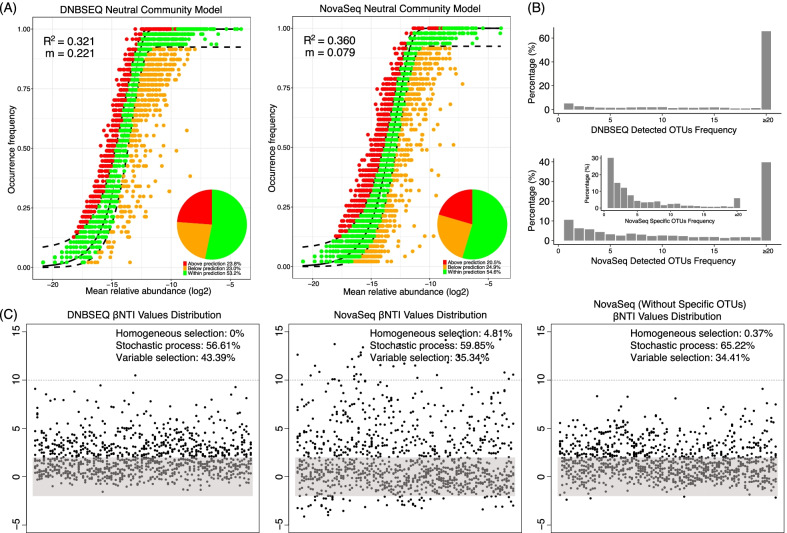
Cow rumen microbial community assembly mechanism. **A** The fit of Sloan’s neutral model to the cow rumen microbial communities revealed by each sequencing platform. The proportion of OTUs distributed within the Sloan’s neutral model, at the upper or lower part were indicated. R^2^ values indicate the overall fit of the model, and m values indicate the estimated migration rate. Dashed lines represent 95% confidence intervals around the model prediction. **B** Bar charts shows the OTUs’ frequency distribution on each sequencing platform. More singleton OTUs and low frequent OTUs were observed on NovaSeq sequencing platform, which was largely driven by the OTUs specifically detected by NovaSeq platform. **C** The distribution of βNTI values between cow rumen microbial communities revealed by each sequencing platform. Each point represents a βNTI value. A |βNTI| value of less than 2 (grey shaded region) indicates stochastic assembly processes; a βNTI value of less than − 2 indicates a homogeneous selection event; and a βNTI value of greater than 2 indicates a heterogeneous selection event

The higher diversity of the metacommunity could be attributed to either higher alpha diversity in each sample or higher beta diversity among samples detected by NovaSeq platform. Comparison of the alpha diversity revealed by different sequencing platforms indicated significantly lower Chao I index for NovaSeq platform. But higher phylogenetic diversity was observed for NovaSeq dataset (although not significant), and its unique rare taxa spanned a wider range of the phylogenetic tree than DNBSEQ dataset although their Chao I index was significantly lower (Fig. 3B). Frequency analysis of the NovaSeq specific rare OTUs showed that more than 30% of them were detected only once across all the samples, consistent with the observed higher beta diversity (Fig. 4B, Additional file 7: Fig. S6) and higher diversity of the metacommunity revealed by NovaSeq (Fig. 3A). Taxonomic annotation of the OTUs revealed six phyla (Armatimonadetes, BRC1, Chloroflexi, Deinococcus thermus, Genmatinonadetes, candidate division WPS-2) exclusively detected by NovaSeq, all of which were not commonly reported microbes of cow rumen system (Fig. 3C).

In order to further evaluate whether these NovaSeq specific rare taxa were *bona fide* rare taxa or false positive introduced during sequencing, we assessed the potential correlations between each rare taxa and a set of physiochemical properties of the rumen fluid. The hypothesis was that *bona fide* rare taxa in cow rumen should be correlated with the fermentation condition of their host with a higher probability than randomly introduced false positives. We calculated the correlation between rare taxa detected by each sequencing platform respectively and a set of physiochemical parameters, including the relative concentration of NH4^+^, acetate, propionate, butyrate and iso-butyrate. Consistently higher fractions of rare taxa reported by DNBSEQ platform were found to be significantly correlated with each of physiochemical parameters compared to rare taxa detected by NovaSeq platform (Additional file 1: Table S5), further suggesting that the NovaSeq platform might detect higher fraction of false positive rare taxa than DNBSEQ.

### Index misassignment could lead to biased community assembly mechanisms

The assembly of microbial communities in various ecosystems was simultaneously controlled by both stochastic and deterministic processes with each of them governing a differential fractions of the microbial community compositions in different ecosystems [1, 39, 40]. Understanding the mechanisms of microbial community assembly process is vital for microbiome intervention for host health management [41]. However, how the large amount of potential false positives and missed *bona fide* rare taxa would influence the interpretation and understanding of the mechanism behind community assembly remained elusive. In order to answer this question, both Sloan’s neutral community model [42] and the null assembly model [43, 44] were applied to the cow rumen microbiome data sets generated on both sequencing platforms to investigate: 1) whether stochastic or deterministic process dominated the assembly process of the cow rumen microbiome; 2) whether similar or distinct assembly mechanisms would be revealed by different sequencing platforms.

Neutral model gave relatively low coefficients of the neutral fit (R^2^ = 0.321 for DNBSEQ; R^2^ = 0.360 for NovaSeq) for microbial communities, and the coefficient revealed by both sequencing platforms was comparable (Fig. 4A). Less than 55% of the OTUs distributed within the neutral prediction, indicating that the neutral process could only explain limited part of the microbial community assembly process in cow rumen. The estimated migration rate, *m*, is widely used as an indicator of the probability that a random loss of an individual in a local community would be replaced by dispersal from the metacommunity, as opposite to reproduction within the local community. The value of m was larger on DNBSEQ-G400 than NovaSeq 6000 platform (m = 0.221 for DNBSEQ; m = 0.079 for NovaSeq), indicating a potential communication of microbes among cohousing cows.

In order to further discriminate between the deterministic and stochastic processes in cow rumen microbial community assembly, we calculated the β-nearest taxon index (β-NTI), which quantifies the difference between the observed phylogenetic turnover between observed and null communities. The fractions of community assembly process explained by stochastic process (|β-NTI|< 2), variable selection (β-NTI ≥ 2) and homogeneous selection (β-NTI ≤ -2) were calculate for each sequencing platform respectively (Fig. 4C). Consistent with the results of Sloan’s neutral model fitting, distribution of β-NTI for both sequencing platforms indicated that the cow rumen microbial community assembly was simultaneously controlled by both stochastic and deterministic forces. Compared to DNBSEQ, NovaSeq platform showed a wider range of distribution of the β-NTI values. And, a subtle sign of homogeneous selection was exclusively observed in the NovaSeq results (Fig. 4C). Removal of NovaSeq specific OTUs from the NovaSeq dataset returned similar β-NTI value distribution pattern as that of DNBSEQ platform (Fig. 4C).

### Differential keystone species were identified by DNBSEQ and NovaSeq sequencing platforms

**Fig. 5:**
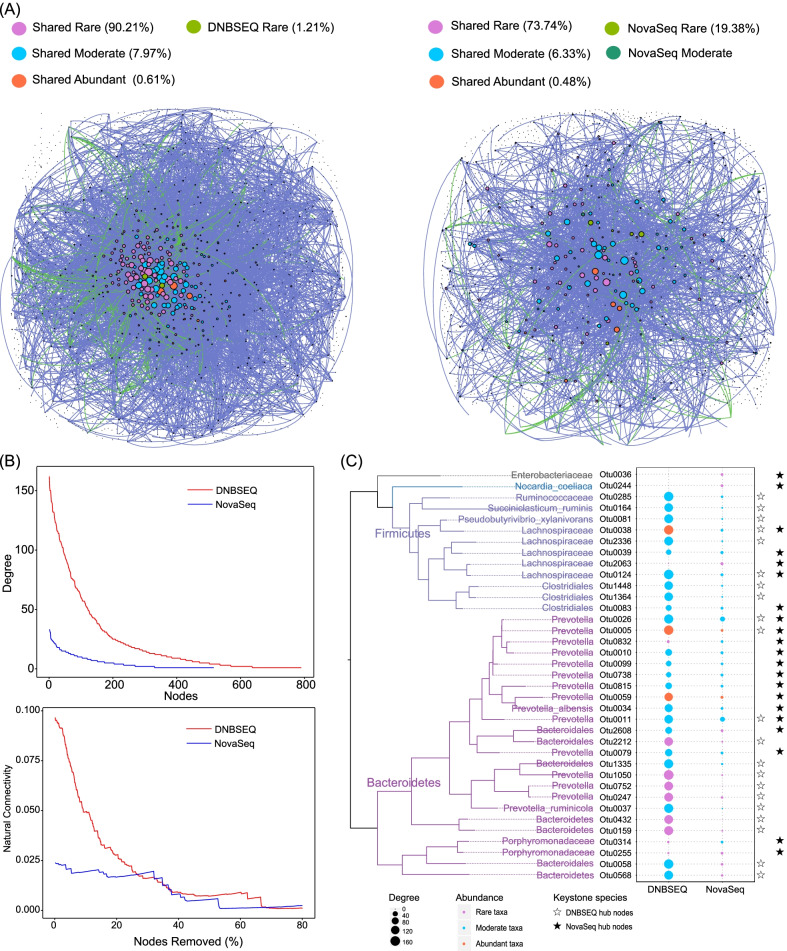
Cow rumen microbial interaction network revealed by DNBSEQ and NovaSeq. **A** The cooccurrence network of microbial communities in cow rumen. Each node represents an OTU and was color coded by both sub-community type and sequencing platform where appropriate. The size of the node is proportional to its node degree. Each line represents a potential correlation interaction, with blue lines indicating positive interaction while green lines indicating negative interaction. Only interactions with a correlation coefficient greater than 0.75 and significance of *P* smaller than 0.05 were plotted. **B** Node degree distribution and the Natural Connectivity change under node attack test of the microbial interaction network revealed by DNBSEQ and NovaSeq platforms. **C** Phylogeny of the top 20 nodes with the highest degree in the microbial interaction networks revealed by DNBSEQ and NovaSeq. The size of the solid circle is proportional to its node degree and color coded by the sub-community type. Solid or open asterisk indicates the platform by which the OTUs were identified as the top 20 nodes with the highest degree

Researchers often infer interactions among microbes based on the correlation coefficients of their relative abundances distributed in a set of samples. The architectural or topological features of networks could provide invaluable insights into complex polymicrobial interactions and co-occurrence patterns, and could be used to identify microbes playing the most influential roles in the community, such as keystone species. Thus, we assessed whether potential false positives may lead to misleading or even wrong interpretations of microbial interactions. Microbial interaction network for rumen microbial communities revealed by each sequencing platform was constructed based on the co-occurrence correlations (Fig. 5A). Rare taxa contributed more than 90% of the nodes for each network, demonstrating potential important ecological roles of the rare taxa in cow rumen. The degree of nodes of both networks followed a power-law distribution, showing the property of scale-free networks. However, the microbial network based on NovaSeq platform was less integrated with significant lower node degree and stability under node attack compared with the network based on DNBSEQ platform (Fig. 5B). Although microbes from Lachnospiraceae, Clostridiales, Bacteroidales and *Prevotella* were identified as keystone species by both sequencing platforms, closely related but distinct OTUs from each of these taxa were identified (Fig. 5C), which might be caused by minor sequencing biases or errors of different sequencing platforms leading to the formation of different OTUs with minor differences during clustering process using 100% sequencing identity algorithm. This minor within genus or even species difference would not lead to wrong interpretation of the ecological roles of these microbes. However, two of the keystone species with high node degree identified by DNBSEQ, including *Pseudobutyrivibrio xylanivorans* (Lachnospiraceae) and *Succiniclasticum ruminis* (Acidaminococcaceae), both of which were reported to play important ecological roles in rumen system [45, 46], did not occupy a hub position in the interaction network based on NovaSeq sequencing results. On the contrary, several of keystone species identified by NovaSeq platform were with low degree (such as Porphyromonadaceae and Enterobacteriaceae) or not detected (*Nocardia coeliaca,* Otu0244) by DNBSEQ platform. While there were previous publications reporting the observation of Porphyromonadaceae, Enterobacteriaceae in cow rumen ecosystem [47–49], *Nocardia coeliaca* was reported as an aerobic, gram-positive bacterial and not a frequently observed microbe in cow rumen [50]. PCR verification of *Nocardia coeliaca* using specifically designed primers were conducted, and Sanger sequencing of the weak positive clones returned low sequencing identity (~ 95%) to the target *Nocardia coeliaca* representative OTU sequence (Additional file 1: Table S6).

## Discussion

The past decade has seen a rising interest and understanding of the microbial rare biosphere [51–53]. In this study, we carefully evaluated the rate of potential index misassignment of two main stream sequencing platforms based on different sequencing technologies, and found significant higher fractions of unexpected reads for the NovaSeq 6000 platform. Although the unexpected reads might be introduced during library construction from neighboring wells as described previously [33], neighboring well might not be enough to explain the observed high phylogenetic diversity and the large fraction of the unexpected taxa. Lower mapping rate of the shotgun sequencing reads of the same samples to their respective NovaSeq OTUs also support the possibilities of potential contaminations caused by index misassignment. Furthermore, the fraction of unexpected reads in the mock community test was consistent with previous report demonstrating up to 6% index misassignment on Illumina sequencing platforms [23, 28]. Taking together, it might be reasonable to infer index misassignment might be the major cause of those observed false positives.

Tools and algorithms for potential contamination removal have been developed previously, such as Decontam [54] and PERFect [55] regarding amplicon sequencing data. While Decontam removes likely abundant contaminant taxa based on statistical test but does not address the false positive issue of rare taxa, PERFect just removes rare taxa and demonstrates that rare taxa removal would not influence the overall statistical results of microbial communities [56], both of which were not suitable to remove index misassignment caused false positives, especially when rare taxa were focused. Moreover, arbitrarily removing reads with small number of copies in a dataset could harm the study of *bona fide* rare taxa, although the abundant taxa were demonstrated not likely to be influenced [56]. As index misassignment happens in a random way, we assume proper technical replication setting and thorough cross-validation between replicates could partly alleviate the number of false positive OTUs, but caution still should be taken when focusing on the rare biosphere as some *bona fide* rare taxa might be missed and consistent detection in triplicates still did not guarantee the observation of *bona fide* rara taxa when NovaSeq sequencing platform was used and all technical replications were sequenced in the same run (Fig. 1, Additional file 3: Fig. S2, Additional file 4: Fig. S3).

Amplicon sequencing test of microbial communities from three real ecosystems with differential complexity confirmed significant batch effects that were probably caused by index misassignments especially for the rare sub-community. There are also previous works from other peer colleagues demonstrating significant but rarely considered run-to-run variations in microbial community studies using amplicon sequencing technology [51, 57–59]. Special caution should be taken when sequencing low biomass samples, as the low biomass samples were more vulnerable to index misassignment when pooled and sequenced with high biomass samples due to imbalanced index usage [34].

Index misassignment could also lead to inflated alpha diversity for relatively simple ecosystems but lower estimated alpha diversity for more complex samples. Because given certain sequencing depth, it is easier to recover all the microbial taxa in relatively simple communities, and the successful detection of rare taxa would less likely be affected by index misassignment, leading to higher estimation of alpha diversity. However, for complex microbial community in complex ecosystems, such as mangrove sediment and cow rumen, more *bona fide* rare taxa would be contained, the successful detection of which could be more easily diluted out by highly occurred false detections, leading to lower observed alpha diversity. Technical replications in this case could hardly improve the estimation of true alpha diversity as *bona fide* rare taxa’s omission and false positive’s introduction were stochastic (Fig. 2B).

In addition to inflated or underestimated alpha diversity and biased microbial compositions, index misassignment introduced false positive rare taxa could affect the interpretation of microbial community assembly mechanism and identification of keystone species (Fig. 5).

Regarding community assembly mechanism, the overall low fit of neutral model by both platforms was easy to understand as the cow rumen environment should exert certain selective pressure on its microbiome, which was in accord with previous study demonstrating that age and diet played an overall deterministic force over the entire microbial community after a stochastic microbial colonization at birth [60]. However, the migration rate m revealed by each sequencing platform were quite different. A migration rate m = 1 indicated an entirely open and highly coupled local and metacommunity, while a migration rate of 0 indicated an entirely isolated local community. With the drop of migration rate, the internal neutral dynamics increasingly act to dominant the dynamics of the local community until totally control and make the local community isolated [42, 61]. Thus the extremely low migrate rate revealed by NovaSeq platform might not fit the case in our study as all the samples were from cows raised under the same controlled husbandry regimes, diets and conditions as discussed above, and there should be substantial exchange of the rumen microbiome considering the co-housing and the natural rumination process [60]. The low migration rate might be explained by the large fraction of singleton and very low frequent OTUs specifically detected by NovaSeq platform (potential false positives) (Fig. 4B), because the loss of singleton or low frequent OTUs could not or hardly be filled by an “immigrant” from the metacommunity, leading the local community more “isolated”. The overall lower fraction of microbial taxa correlated with various key rumen physiochemical properties also suggested less overall confidence of the NovaSeq detected rare taxa. The NovaSeq platform specifically observed homogeneous selection process (β-NTI ≤  − 2) might be attributed to the homogeneously introduced potential false positives by index misassignment, as revealed by the NovaSeq specific OTUs with high frequency. The NovaSeq platform observed very large β-NTI values (β-NTI ≥ 10), representing variable deterministic selection process, might be caused by randomly introduced differential false positives from other non-relevant samples processed on the same sequencing lane, as revealed by the higher phylogenetic diversity. Although very large β-NTI values were reported by NovaSeq dataset, NovaSeq revealed less overall fraction of variable selection process compared to DNBSEQ, which might be because a fraction of differential *bona fide* rare taxa was missed by NovaSeq platform as revealed by the lower estimated alpha diversity compared to DNBSEQ.

Various microbes in a community interact with each other to communicate, cross-feed, recombine, and coevolve, in a way via which microbes form a complex interaction network and sustain their stability and robustness [62]. The cow rumen microbial interaction networks based on both sequencing platforms revealed that rare taxa occupied most of the nodes and some of them were even identified as keystone taxa, consistent with previous work demonstrating keystone species in a community were not necessarily to be dominant [9]. However, differential taxa were identified by each sequencing platforms, which should be interpreted with cautions. For example, the NovaSeq identified keystone species, *Nocardia coeliaca*, was documented as an aerobic soil bacteria [50] and confirmed negative from the rumen genomic DNA sample using specifically designed PCR primer pairs. Keystone species could be the most influential microbes in a network interacting with most other microbes and essential for the stability and robustness of the microbial community [62]. Wrong identification of keystone species thus could lead to very miss-leading interpretation of the potential ecological roles of certain microbes and even wrong understanding entire microbial community.

## Conclusions

In amplicon studies, although index misassignment would not have significant influence to the relative abundant taxa, it could lead to biased features regarding the rare sub-community, including their composition, diversity, interaction network, assembly mechanisms and other properties to be found. Potential contaminants could also be introduced from any of the experimental processes, including extraction, PCR amplification, library construction and other processes. As index misassignment happens in a random way, we assume proper technical replication setting and thorough cross-validation between replicates could partly alleviate the number of false positive OTUs, but caution still should be taken when focusing on the rare biosphere as some *bona fide* rare taxa might be missed and consistent detection in triplicates still did not guarantee the observation of *bona fide* rara taxa when NovaSeq sequencing platform was used and all technical replications were sequenced in the same run. Properly set positive and negative controls, including blank extraction kit, together with proper quality control and bioinformatic algorithms during data processing, could also be used to eliminate the potential contaminants. Furthermore, proper sequencing platforms with low potential index misassignment rate and enough sequencing depth are suggested to improve the accuracy of rare taxa detection and downstream biological and ecological mechanisms interpretation. We also recommend researchers to cross validate the metabarcoding amplicon sequencing results using differential sequencing technologies when focusing on rare sub-community study.

## Materials and methods

### Mock communities, typical sample collection and DNA extraction

The commercial mock microbial community, ZymoBIOMICS™ Microbial Community DNA Standard D6305, containing 8 bacteria strains was purchased from Zymo Research. Theoretical compositions of the ZymoBIOMICS™ could be found from its official instructions, and the theoretical bacterial compositions were provided in supplementary table (Additional file 1: Table S1). Two customized mock communities, one of which containing 4 bacterial strains (genomic DNA of *Bacillus halotolerans*, *Bosea robiniae*, *Streptomyces toxytricini* and *Nocardiopsis dassonvillei* mixed in a ratio of 2:1:1:1), and the other containing 7 bacterial strains (genomic DNA of *Photobacterium halotolerans, Vibrio parahaemolyticus, Vibrio natriegens, Bacillus aquimaris, Bacillus anthracis, Bacillus aryabhattai* and *Bacillus hwajinpoensis* mixed in a ratios of 10:10:10:10:1:1:1) were constructed. All the bacteria strains used in the customized mock communities were obtained from China National GeneBank (Qingdao), BGI-Qingdao, China.

Three mice fecal samples were selected from the deposited samples at China National GeneBank (Qingdao, China) in August, 2019, and total DNA was extracted using QIAamp DNA Stool Mini Kit (Qiagen, Hilden, Germany). Three surface seawater filter samples were collected from Wentai Fishery (Zhejiang, China) by filtering 2L of surface seawater in April, 2018, and the filter samples were used for total DNA extraction using the DNeasy Blood and Tissue Kit (Qiagen, Hilden, Germany) [63]. Three mangrove rhizosphere topsoil samples were collected from East Harbour National Nature Reserve (Hainan, China) in April, 2018, and 0.5 g of each soil sample was used to extract the total DNA using the PowerSoil DNA isolation kit (Mobio Labs, Inc., Solana Beach, CA, USA).

### Cow rumen sample collection, DNA extraction and physiochemical parameters measurement

The lactating Holstein dairy cows with similar age and raised under the same controlled husbandry regimes, diets, and rearing conditions were selected at a commercial dairy farm (Yangling, Shanxi, China). Rumen fluid were collected from cows via esophageal tubing before morning feeding. Firstly, several hundreds of milliliters of rumen fluid were discarded to minimize saliva contamination. Then the rumen fluid samples were filtered through four layers of cheesecloth, and stored at -80 °C before DNA extraction. Rumen fluid was centrifuged at 12,000 × g for 10 min at 4 °C for supernatant collection. Total DNA was extracted from the centrifuged pellet using a method involving cetyltrimethylammonium bromide (CTAB) plus bead beating (Minas et al., 2011). Ruminal supernatant was used for volatile fatty acids analyzation by gas chromatography (Agilent 7890A, Wilmington, USA). The NH_3_-N concentration in supernatant was determined using a Berthelot ammonia assay kit (Jiancheng, Nanjing, China).

### PCR Amplification, library construction and sequencing

The universal primer pair for 16S rRNA gene V4 region 515F/806R (515F: GTGCCAGCMGCCGCGGTAA, 806R: GGACTACHVGGGTWTCTAAT) [64] were used for PCR amplification. Triplicate PCR reactions, library constructions, and sequencing runs were carried out to evaluate the reproducibility and potential batch effects of each sequencing platform. Both positive and negative PCR controls were included in the PCR amplification step. Each of the PCR products was subject to library construction flowing the instructions of the respective sequencing platforms. Negative controls of PCR were failed in library preparation and only successfully constructed libraries were subjected to following metabarcoding and sequencing procedure. A two-step PCR procedure was used to construct the amplicon libraries to be sequenced at the DNBSEQ-G400 sequencing platform as previously described [65]. Basically, for all samples and negative controls, the first-step PCR with zero to three random nucleotides inserted before each of the primer pairs to balance nucleotide proportion at each position for accurate base-calling was performed as follows: 95 °C for 10 min, followed by 20 cycles at 98 °C for 20 s, 58 °C for 30 s, and 72 °C for 30 s with a final extension at 72 °C for 10 min. No target PCR product band was observed for the negative PCR controls. After the amplification, the primer with sample barcode and the DNBSEQ sequencer adapter was used for the second PCR amplification: 95 °C for 5 min, followed by 15 cycles at 98 °C for 20 s, 58 °C for 30 s, and 72 °C for 30 s with a final extension at 72 °C for 10 min. After the two-step PCR amplification, the PCR products were verified using 1.5% agarose gel electrophoresis. The PCR products with target bands were mixed in equal mass, and 2% agarose gel was used for electrophoresis and gel cutting for purification, and then make DNA nanoballs (DNB) following the standard protocol of the DNBSEQ sequencing platform. All libraries were sequenced on DNBSEQ-G400 platform in the paired-end mode with 200 bp length reads at BGI-Qingdao (Qingdao, China).

For the Illumina amplicon sequencing library construction, about 10 ng DNA for each sample was used for the PCR amplification using the 515F/806R 16S rRNA gene primer pair. The PCR procedure was as follows: 98 °C for 1 min, followed by 30 cycles of denaturation at 98 °C for 10 s, annealing at 50 °C for 30 s, and elongation at 72 °C for 30 s with final extension at 72 °C for 5 min. PCR products with target bands were mixed in equal mass, and then the mixed PCR products were purified with GeneJET Gel Extraction Kit (Thermo Scientific). Sequencing libraries were constructed using Illumina TruSeq DNA PCR-Free Library Preparation Kit (Illumina, USA) following the manufacturer’s protocol with index sequence and then libraries quality was assessed by Qubit 2.0 Fluorometer (Thermo Scientific). For all technical triplicate of mocks and typical samples, the qualified libraries were sequenced on an Illumina NovaSeq 6000 platform and generated 250 bp paired-end reads using the same sequencing provider Novogene Co., LTD (Beijing, China), to eliminate confounding bias introduced by different laboratories. Cow rumen samples were sequenced using the same library construction and sequencing strategies using a different sequencing provider Personalbio Co., LTD (Shanghai, China), to test whether higher fraction of potential false positives would be reported by another sequencing provider. No technical replication was set for cow rumen samples as most researchers do when sequencing large number of samples.

### Quality control of reads and the bioinformatical process

An average of 50,000 ~ 60,000 reads were generated for each of the samples in the present study. Reads with adapter contaminations and low-quality reads (more than 20% base quality < Q20) in the raw data set were filtered out by SOAPnuke (v1.5.6) [66]. Paired-end high quality clean reads were merged into tags by FLASH (v1.2.11) [67] with parameters “–min-overlap 10 –max-mismatch-density 0.1”. Reads generated from different sequencing platforms were combined and the denoising clustering algorithm unoise3 [20] was used to generate denoised OTUs by USEARCH (v10.0.240) [68] with default parameter of “-minsize 8”, and generated the OTU abundance profiles [69]. Another denoising clustering algorithm DADA2 (version 1.20.0) was also used to generate exact amplicon sequencing variants (ASVs). As two denoising algorithms gave highly similar results (Additional file 8: Fig. S7) regarding both community composition and diversity. Since there were already papers comparing these different algorithms [21], and comparison of algorithms was out the scope of the current study, we used the results of unoise3 for all the following analysis. OTU taxonomic assignment was carried out using sintax algorithm [70] against RDP training set (v18) with 0.8 confidence cutoff value. The phylogenetic tree of cow rumen OTUs was constructed by FastTree (v2.1.5) [71] and visualized by iTOL [72]. The alpha-diversity index including Shannon indices and Chao I indices, weighted and unweighted UniFrac beta-diversity distances were analyzed using QIIME (v1.9.1). The phylogenetic diversity index was calculated using R package “picante”. All boxplots were visualized by R package “ggplot2”. The venn diagrams were plotted by R package “venn” and software TBtools (v1.09856). To determine the potential false positive taxa, more than 150 Gb metagenomics sequencing data [35] of the three mangrove rhizosphere soil samples, and 15 Gb metagenomics sequencing data of mice gut samples (data not published) were mapped to the mangrove or mice gut represent OTUs, respectively, using Salmon [73] (v0.9.1). The OTUs with more than one metagenomic read mapped in average were masked as OTUs consistently detected in shotgun sequencing.

### PCR verification of Nocardia coeliaca in rumen fluid

Specific primer pairs for amplification of *Nocardia coeliaca* were designed based on the representative sequence of Otu0244 using primer premier (V6.0). One forward and two reverse primers were designed with their sequences as follows: Otu0244_F1: AGGCGGTTTGTCGCGTCGTT, Otu0244_R1: TCGCTACCCACGCTTTCGTTCC; and Otu0244_R2: ACGCTTTCGTTCCTCAGCGTCA. The genome DNA from three different rumen fluid samples were mixed by equal mass and used for PCR amplification to verify the presence or absence of *Nocardia coeliaca* in the cow rumen ecosystem. The representative sequence of Otu0244 was directly synthesized in Sangon Biotech (Shanghai, China), and was used as positive control for PCR amplification to test the efficiency of the designed primer pairs. PCR products were linked to pESI-T vector and transformed into DH5α competent *E. coli* cells. Positive clones were then picked and sequenced using ABI 3730XL. Sequences of the positive clones were aligned to the entire OTU representative sequences set using blast to search for potential positive hits.

### Statistical analysis

All statistical analysis was conducted in R environment (v3.4.1). Microbes with an averaged relative abundance (RA) of RA ≥ 1% across all the replications were defined abundant taxa and divided into the abundant sub-community; microbes with an averaged RA of 1% > RA ≥ 0.1% were defined moderate taxa and divided into the moderate sub-community; while microbes with an averaged RA of RA < 0.1% were defined as rare taxa and divided into the rare sub-community. The significance test of differential alpha diversity or phylogenetic diversity was assessed by the Wilcoxon-test, while difference between weighted and unweighted UniFrac distances was tested with PERMANOVA using “vegan” Pacakge. Potential correlation between cow rumen OTU relative abundances and the physiochemical properties of the rumen fluid was measured by “spearman” function with a significance cut-off of *P*-value < 0.05.

To compare the assembly mechanisms of cow rumen microbiomes revealed by DNBSEQ or NovaSeq sequencing platforms, both Sloan’s neutral model and the null model hypothesis were tested. The Sloan neutral community model prediction and statistics were performed by “MicEco” package in R [74], and the overall fitness of the model (*R*^*2*^ value) and the proxy of dispersal limitation (estimated migration rate, *m* value) were calculated at the same time [42]. The beta Nearest Taxon Index (βNTI) values, an index representing the null assembly hypothesis, were calculated by “picante” package [75] in R. To assess how NovaSeq specific OTUs, representing potential false positives, could influence the community assembly process, the NovaSeq specific OTUs were removed and the NovaSeq-without-specific OTU βNTI values were calculated compared with DNBSEQ results.

The co-occurrence networks of the microbiomes revealed by DNBSEQ and NovaSeq were preformed respectively using SparCC algorithm [76], and only the robust correlations with |*r|*> 0.75 and *P* < 0.05 were considered. The networks were visualized, and the node degrees were calculated by Gephi (0.9.2) [77]. The natural connectivity of networks was estimated by “attacking” nodes to confirm the robustness of the correlation networks [78].

## Supplementary Information

**Additional file 1: Table S1.** OTU table of the amplicon sequencing result of the ZymoBIOMICS™ Microbial Community DNA Standard. Table S2. OTU table of the customized mock community with four bacteria. Table S3. OTU table of the customized mock community with seven bacteria. Table S4. Summary of the number of reads and clustered OTUs of samples from the three typical ecosystems. Table S5. Correlation test of the relative abundance of OTUs and physiochemical properties of cow rumen fluid. Table S6. Mapping results of the cloned sequences of specifically designed primers for Nocardia coeliaca to the entire OTU representative sequences.

**Additional file 2: Figure S1.** Schematic presentation of the design and flow of this study. This study was designed to evaluate the potential harm of false positives introduced by index misassignment during the sequencing to the interpretation of metabarcoding or here amplicon sequencing studies. In part 1, mock communities with known microbial composition were used to confirm the presence of index misassignment introduced false positives and the rate of this misassignment on different sequencing platform; In part 2, samples from several typical ecosystems with various complexity were sequenced to test how index misassignment could affect the estimation of community diversity for different samples; In part 3, a case study using a set of cow rumen samples was conducted to evaluate how index misassignment caused false positives could affect the interpretation of microbial ecological roles and community assembly mechanisms.

**Additional file 3: Figure S2.** Comparison of shared fraction of OTUs by different sequencing platforms. UpSet graph showing the number of shared OTUs among the technical triplicates of DNBSEQ and NovaSeq sequencing platforms for customized mock community with four (A) or seven (B) microbes used in the current study. And heatmap showing the phylum rank relative abundances revealed by different sequencing platforms for the commercial mock (C) and customized mock communities with four (D) or seven (E) bacteria.

**Additional file 4: Figure S3.** Venn diagrams showing the shared OTUs among distinct samples sequenced within the same batch of sequencing run on the Novaseq platform. The large number of shared OTUs among distinct samples (including two customized communities) were likely sample wise cross contaminations caused by index misassignment.

**Additional file 5: Figure S4.** Comparison of the amplicon sequencing results of three typical ecological systems between DNBSEQ and NovaSeq sequencing platforms. (A) Evaluation of the reproducibility of the amplicon sequencing results of DNBSEQ and NovaSeq sequencing platform in revealing the accumulated relative abundance of microbes of abundant, moderate and rate taxa subcommunity from ecosystems with various complexity (DNB All, DNB 2, DNB 3 denotes number of all unique OTUs detected by DNBSEQ platform, consistently detected by at least 2 technical replicates, and consistently detected by all three technical replicates, respectively. Similar naming scheme was used for the NovaSeq platform). (B) Cross-verification of the OTUs identified by amplicon sequencing on DNBSEQ and NovaSeq platforms and shotgun sequencing results of the same set of mangrove sediment samples. (C) Cross-verification of the OTUs identified by amplicon sequencing on DNBSEQ and NovaSeq platforms and shotgun sequencing results of the same set of mice gut samples.

**Additional file 6: Figure S5.** Weighted (A) and unweighted (B) UniFrac distance-based clustering of the amplicon sequencing results revealed by DNBSEQ and NovaSeq sequencing platforms for samples from the three typical ecosystems.

**Additional file 7: Figure S6.** Weighted (A) and unweighted (B) UniFrac distance-based beta diversity of the cow rumen microbial communities revealed by DNBSEQ and NovaSeq sequencing platforms.

**Additional file 8: Figure S7.** Comparison of the results using unoise3 and DADA2 denoising algorithms. The accumulated OTU relative abundances in each phylum based on unoise3 and DADA2 were graphed as bar-chart side-by-side for all the mock communities and samples from three typical ecosystems.

## Data Availability

The data that support the findings of this study have been deposited into CNGB Sequence Archive (CNSA, https://db.cngb.org/cnsa/) of China National GeneBank DataBase (CNGBdb) with accession number CNP0002138 for the cow rumen samples and accession number CNP0002180 for the mock and typical samples.
