# Supplementary material for: Sequencing introduced false positive rare taxa lead to biased microbial community diversity, assembly, and interaction interpretation in amplicon studies
Source: Environ Microbiome. 2022 Aug 17;17:43. doi: 10.1186/s40793-022-00436-y (PMC9387074; doi:10.1186/s40793-022-00436-y)
Supplement: Supplementary file 2 — Additional file 2: Figure S1. Schematic presentation of the design and flow of this study. This study was designed to evaluate the potential harm of false positives introduced by index misassignment during the sequencing to the interpretation of metabarcoding or here amplicon sequencing studies. In part 1, mock communities with known microbial composition were used to confirm the presence of index misassignment introduced false positives and the rate of this misassignment on different sequencing platform; In part 2, samples from several typical ecosystems with various complexity were sequenced to test how index misassignment could affect the estimation of community diversity for different samples; In part 3, a case study using a set of cow rumen samples was conducted to evaluate how index misassignment caused false positives could affect the interpretation of microbial ecological roles and community assembly mechanisms. [file 40793_2022_436_MOESM2_ESM.pdf]

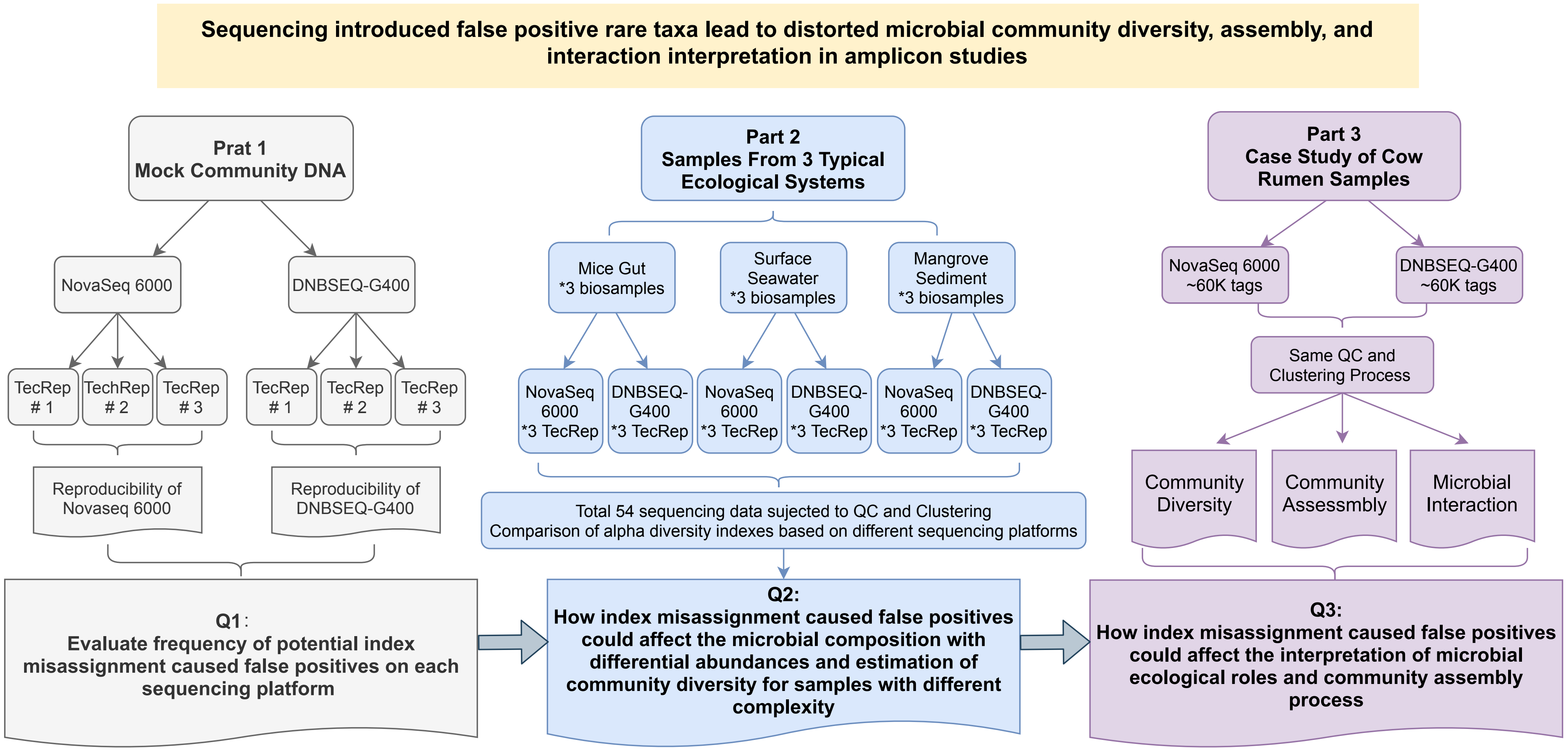
Sequencing introduced false positive rare taxa lead to distorted microbial community diversity, assembly, and interaction interpretation in amplicon studies
Prat 1
Mock Community DNA
NovaSeq 6000
DNBSEQ-G400
TecRep # 1
TechRep # 2
TecRep # 3
TecRep # 1
TecRep # 2
TecRep # 3
Reproducibility of Novaseq 6000
Reproducibility of DNBSEQ-G400
Q1: Evaluate frequency of potential index misassignment caused false positives on each sequencing platform
Part 2
Samples From 3 Typical Ecological Systems
Mice Gut *3 biosamples
Surface Seawater *3 biosamples
Mangrove Sediment *3 biosamples
NovaSeq 6000 *3 TecRep
DNBSEQ-G400 *3 TecRep
NovaSeq 6000 *3 TecRep
DNBSEQ-G400 *3 TecRep
NovaSeq 6000 *3 TecRep
DNBSEQ-G400 *3 TecRep
Total 54 sequencing data sujected to QC and Clustering
Comparison of alpha diversity indexes based on different sequencing platforms
Q2:
How index misassignment caused false positives could affect the microbial composition with differential abundances and estimation of community diversity for samples with different complexity
Part 3
Case Study of Cow Rumen Samples
NovaSeq 6000 ~60K tags
DNBSEQ-G400 ~60K tags
Same QC and Clustering Process
Community Diversity
Community Assessmbly
Microbial Interaction
Q3:
How index misassignment caused false positives could affect the interpretation of microbial ecological roles and community assembly process
