# Supplementary material for: Sequencing introduced false positive rare taxa lead to biased microbial community diversity, assembly, and interaction interpretation in amplicon studies
Source: Environ Microbiome. 2022 Aug 17;17:43. doi: 10.1186/s40793-022-00436-y (PMC9387074; doi:10.1186/s40793-022-00436-y)
Supplement: Supplementary file 3 — Additional file 3: Figure S2. Comparison of shared fraction of OTUs by different sequencing platforms. UpSet graph showing the number of shared OTUs among the technical triplicates of DNBSEQ and NovaSeq sequencing platforms for customized mock community with four (A) or seven (B) microbes used in the current study. And heatmap showing the phylum rank relative abundances revealed by different sequencing platforms for the commercial mock (C) and customized mock communities with four (D) or seven (E) bacteria. [file 40793_2022_436_MOESM3_ESM.pdf]

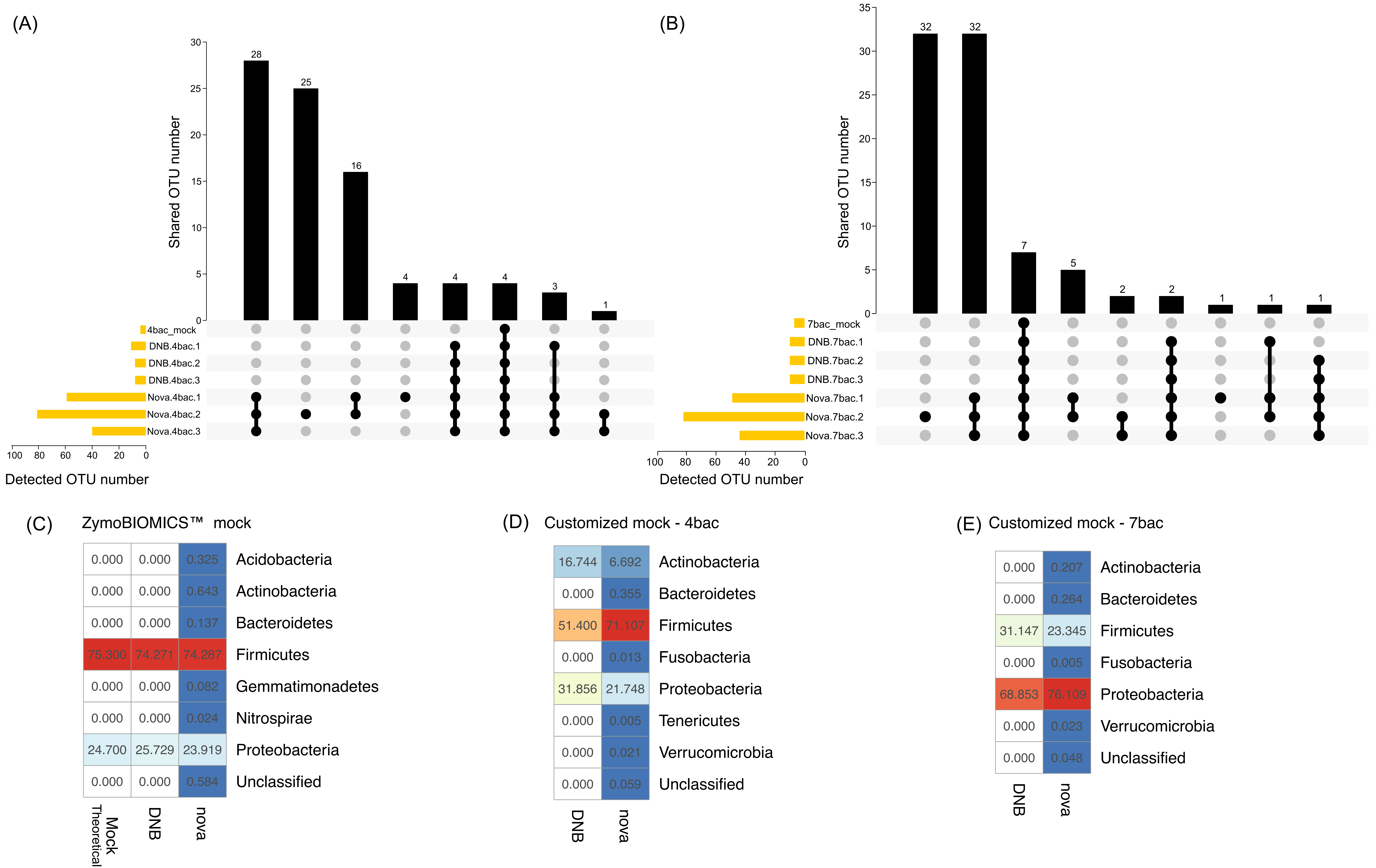
(A)
Shared OTU number
28
25
16
4
4
4
3
1
4bac_mock
DNB.4bac.1
DNB.4bac.2
DNB.4bac.3
Nova.4bac.1
Nova.4bac.2
Nova.4bac.3
Detected OTU number
(B)
Shared OTU number
32
32
7
5
2
2
1
1
1
7bac_mock
DNB.7bac.1
DNB.7bac.2
DNB.7bac.3
Nova.7bac.1
Nova.7bac.2
Nova.7bac.3
Detected OTU number
(C) ZymoBIOMICS™ mock
0.000 0.000 0.325 Acidobacteria
0.000 0.000 0.643 Actinobacteria
0.000 0.000 0.137 Bacteroidetes
75.300 74.271 74.287 Firmicutes
0.000 0.000 0.082 Gemmatimonadetes
0.000 0.000 0.024 Nitrospirae
24.700 25.729 23.919 Proteobacteria
0.000 0.000 0.584 Unclassified
Mock Theoretical
DNB
nova
(D) Customized mock - 4bac
16.744 6.692 Actinobacteria
0.000 0.355 Bacteroidetes
51.400 71.107 Firmicutes
0.000 0.013 Fusobacteria
31.856 21.748 Proteobacteria
0.000 0.005 Tenericutes
0.000 0.021 Verrucomicrobia
0.000 0.059 Unclassified
DNB
nova
(E) Customized mock - 7bac
0.000 0.207 Actinobacteria
0.000 0.264 Bacteroidetes
31.147 23.345 Firmicutes
0.000 0.005 Fusobacteria
68.853 76.109 Proteobacteria
0.000 0.023 Verrucomicrobia
0.000 0.048 Unclassified
DNB
nova
