# Supplementary figures and images for: Sequencing introduced false positive rare taxa lead to biased microbial community diversity, assembly, and interaction interpretation in amplicon studies
Source: Environ Microbiome. 2022 Aug 17;17:43. doi: 10.1186/s40793-022-00436-y (PMC9387074; doi:10.1186/s40793-022-00436-y)

# Batch1

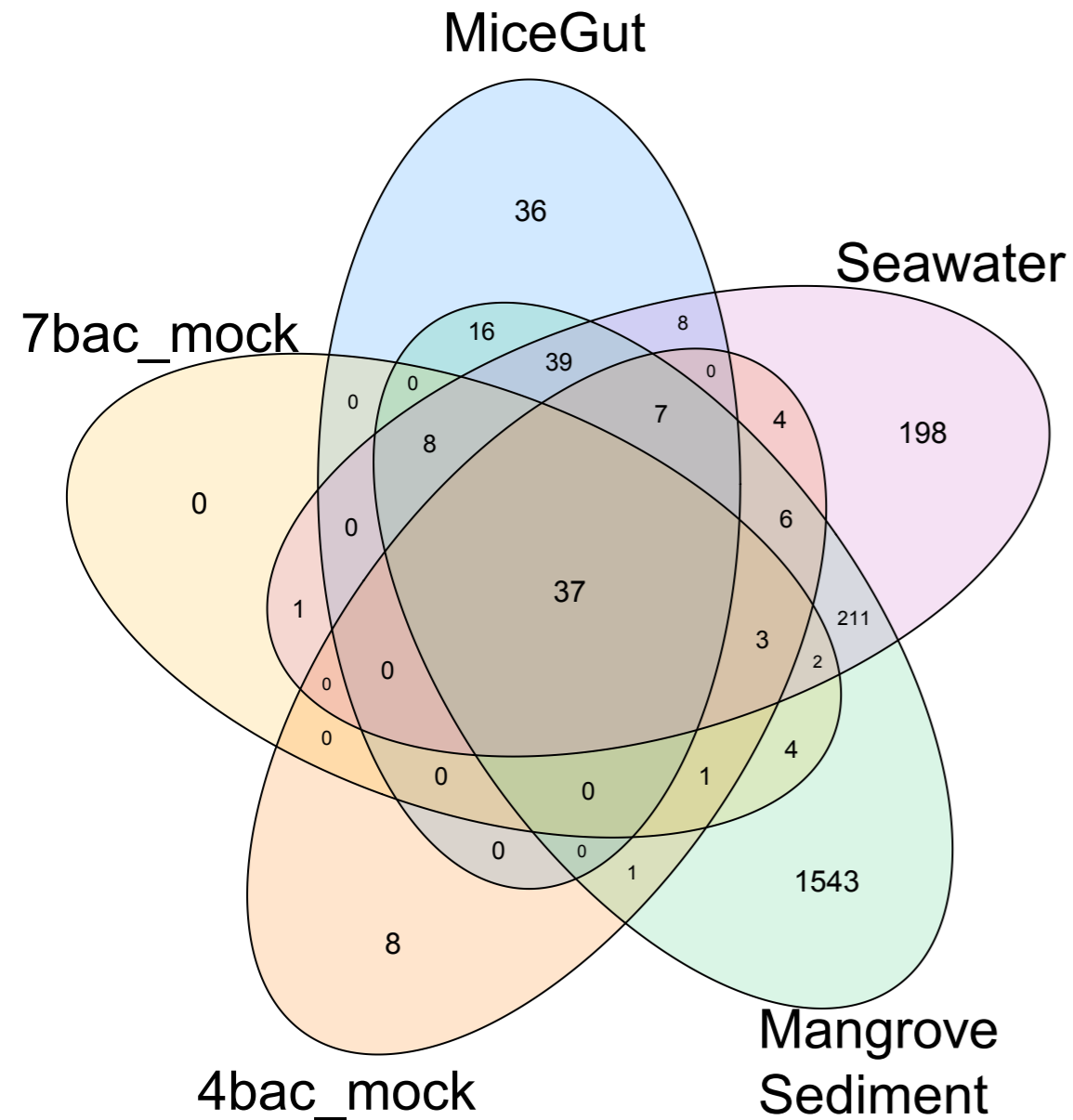

# Batch2

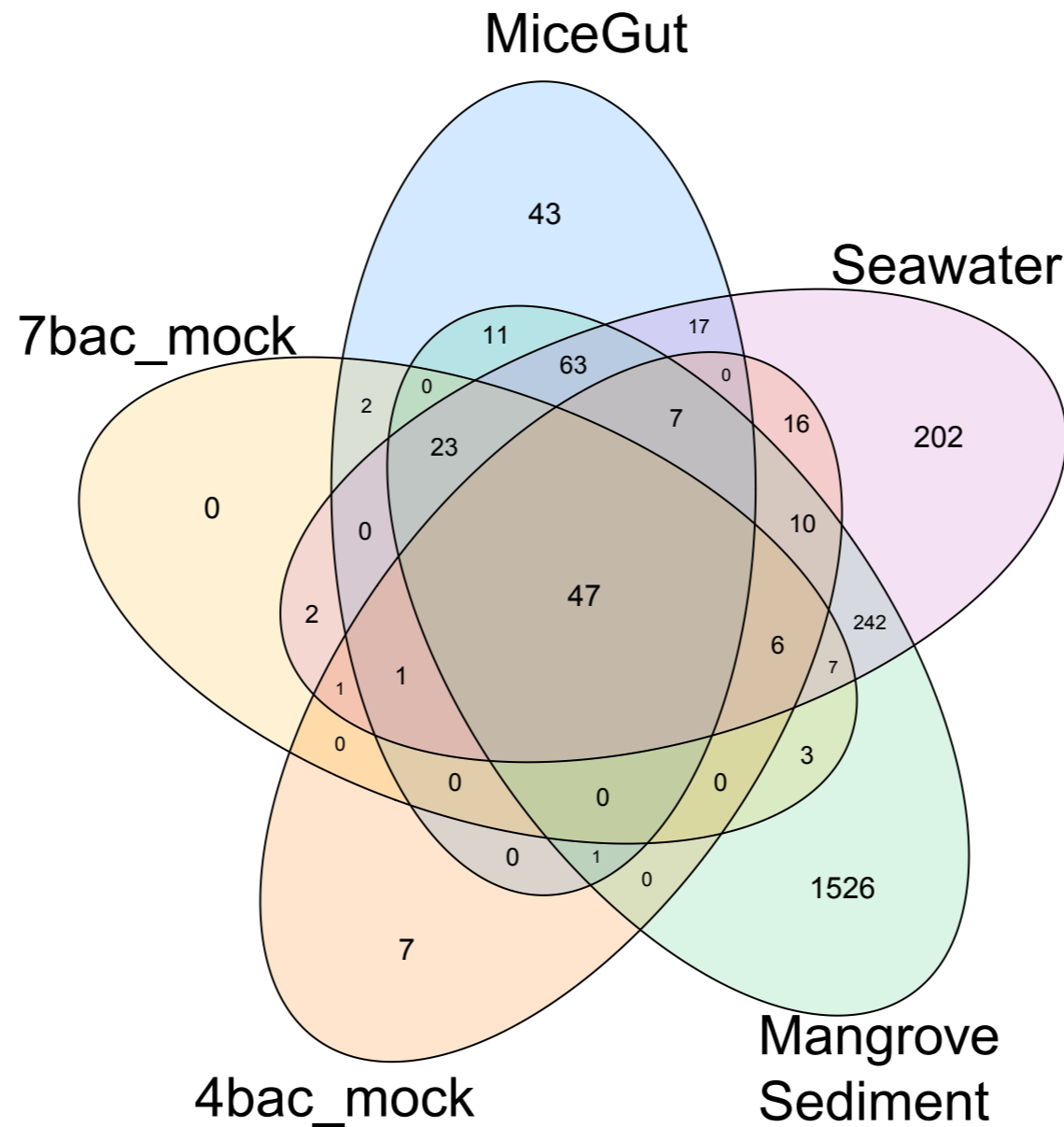

# Batch3

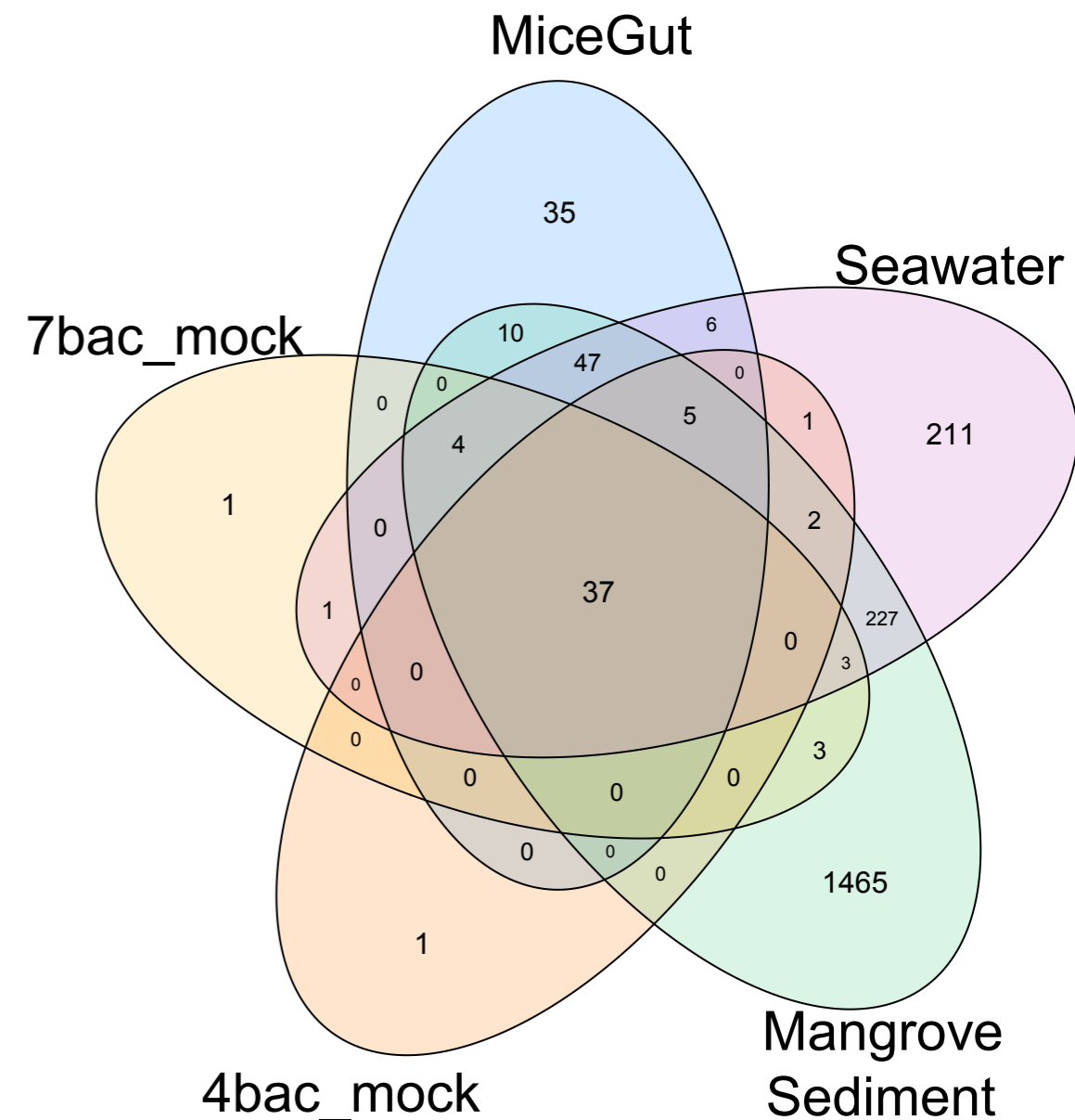

Supplement: Supplementary file 4 — Additional file 4: Figure S3. Venn diagrams showing the shared OTUs among distinct samples sequenced within the same batch of sequencing run on the Novaseq platform. The large number of shared OTUs among distinct samples (including two customized communities) were likely sample wise cross contaminations caused by index misassignment. [file 40793_2022_436_MOESM4_ESM.pdf]

(A) ■ DNBSEQ Specific OTUs ■ NovaSeq Specific OTUs ■ Shared OTUs

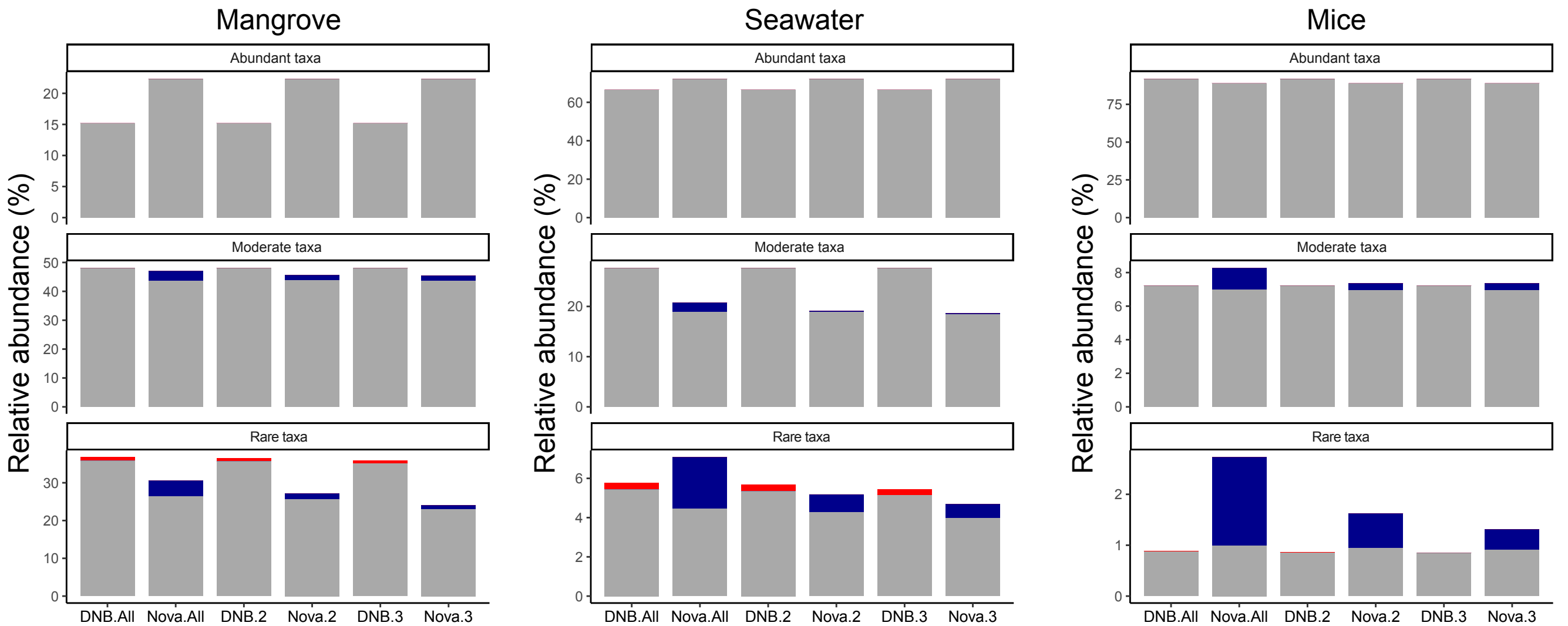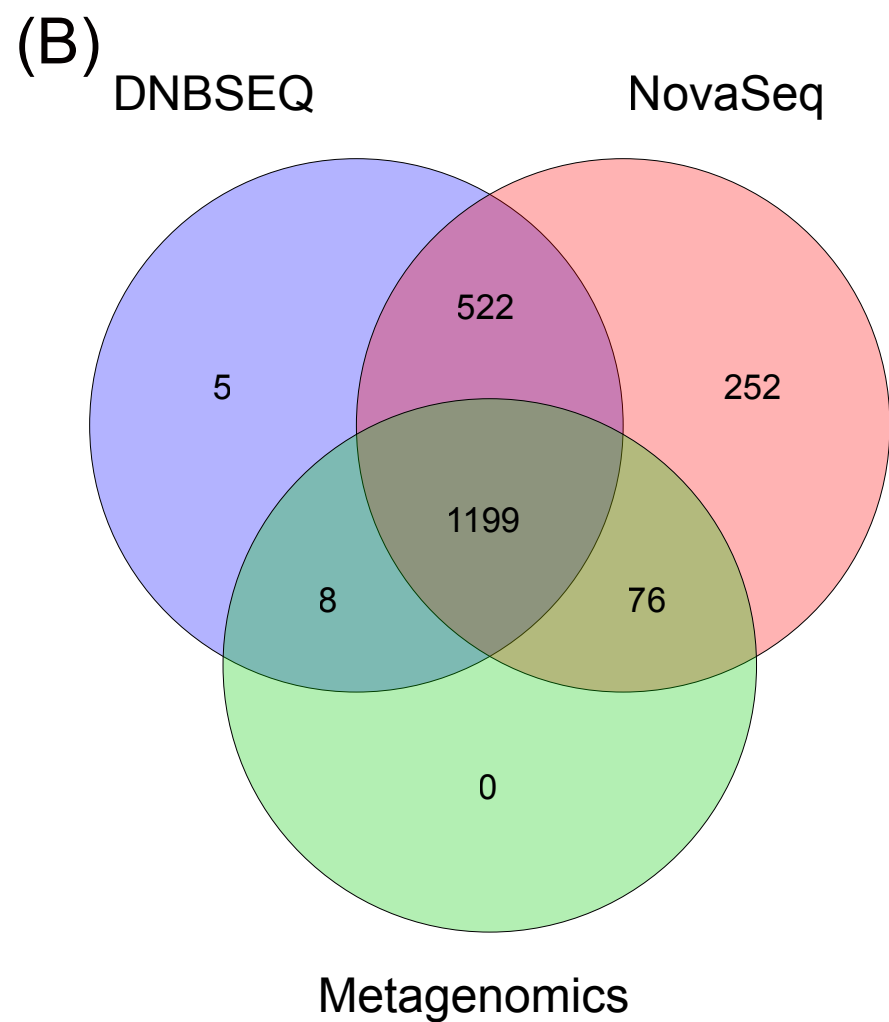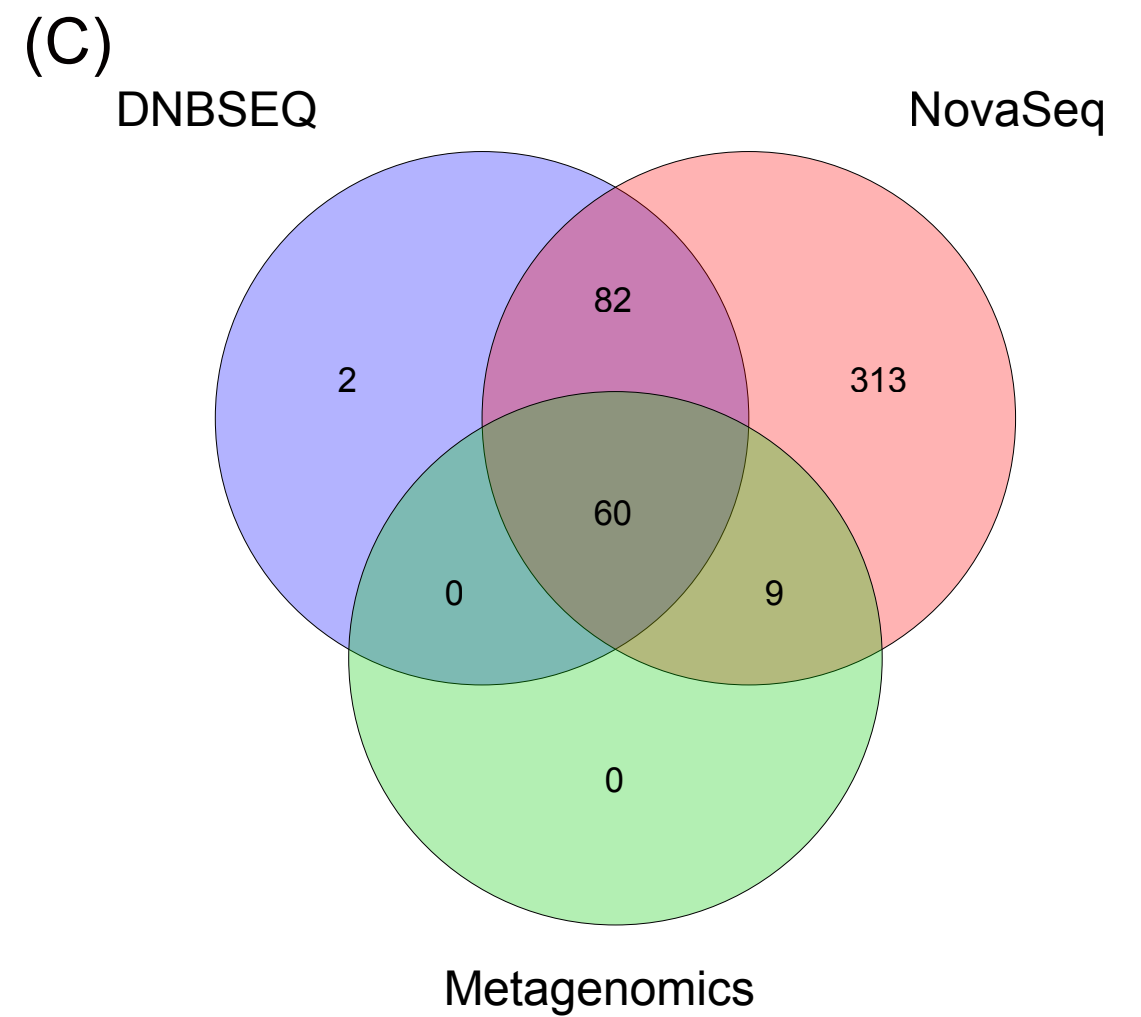

Supplement: Supplementary file 5 — Additional file 5: Figure S4. Comparison of the amplicon sequencing results of three typical ecological systems between DNBSEQ and NovaSeq sequencing platforms. (A) Evaluation of the reproducibility of the amplicon sequencing results of DNBSEQ and NovaSeq sequencing platform in revealing the accumulated relative abundance of microbes of abundant, moderate and rate taxa subcommunity from ecosystems with various complexity (DNB All, DNB 2, DNB 3 denotes number of all unique OTUs detected by DNBSEQ platform, consistently detected by at least 2 technical replicates, and consistently detected by all three technical replicates, respectively. Similar naming scheme was used for the NovaSeq platform). (B) Cross-verification of the OTUs identified by amplicon sequencing on DNBSEQ and NovaSeq platforms and shotgun sequencing results of the same set of mangrove sediment samples. (C) Cross-verification of the OTUs identified by amplicon sequencing on DNBSEQ and NovaSeq platforms and shotgun sequencing results of the same set of mice gut samples. [file 40793_2022_436_MOESM5_ESM.pdf]

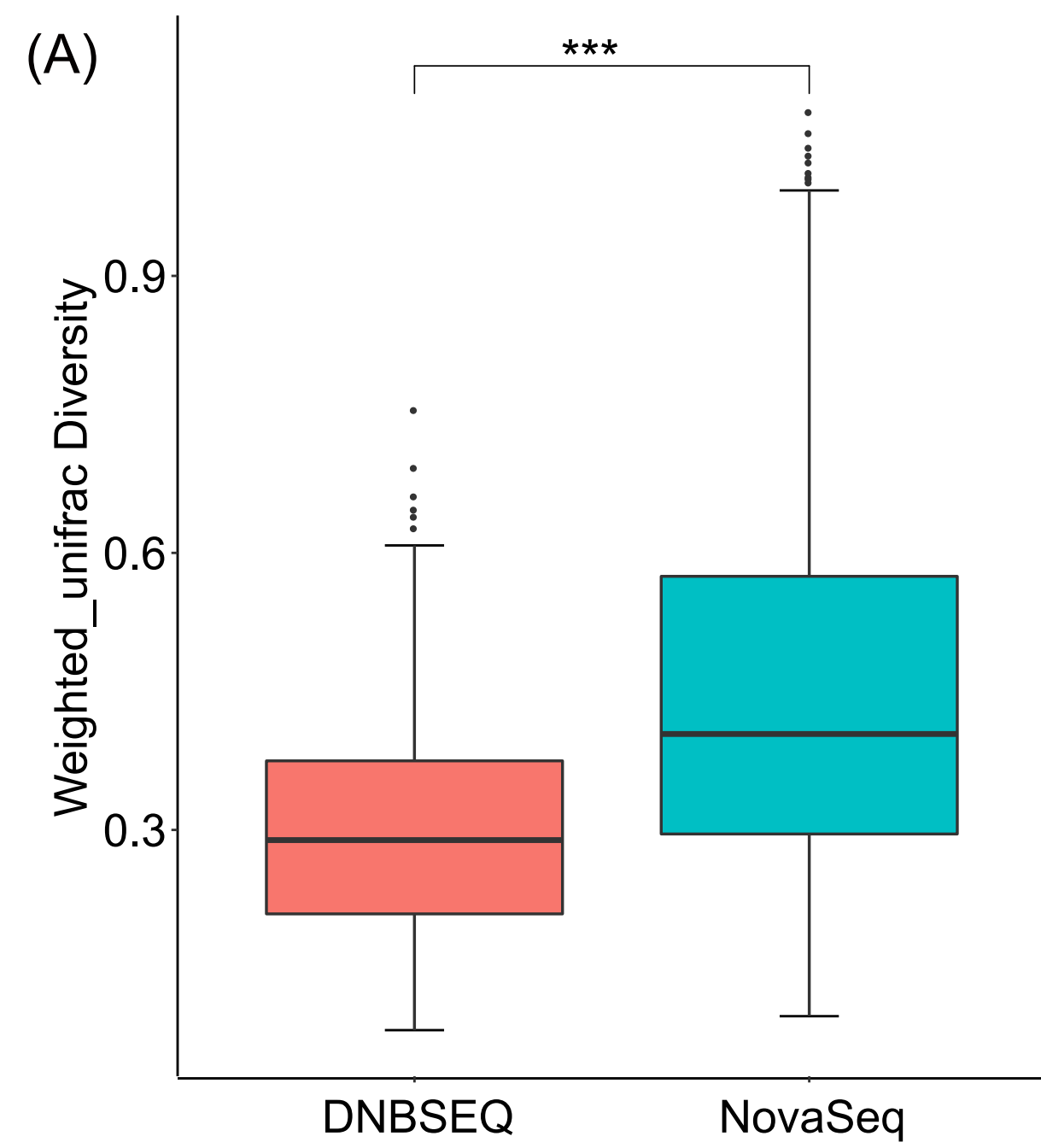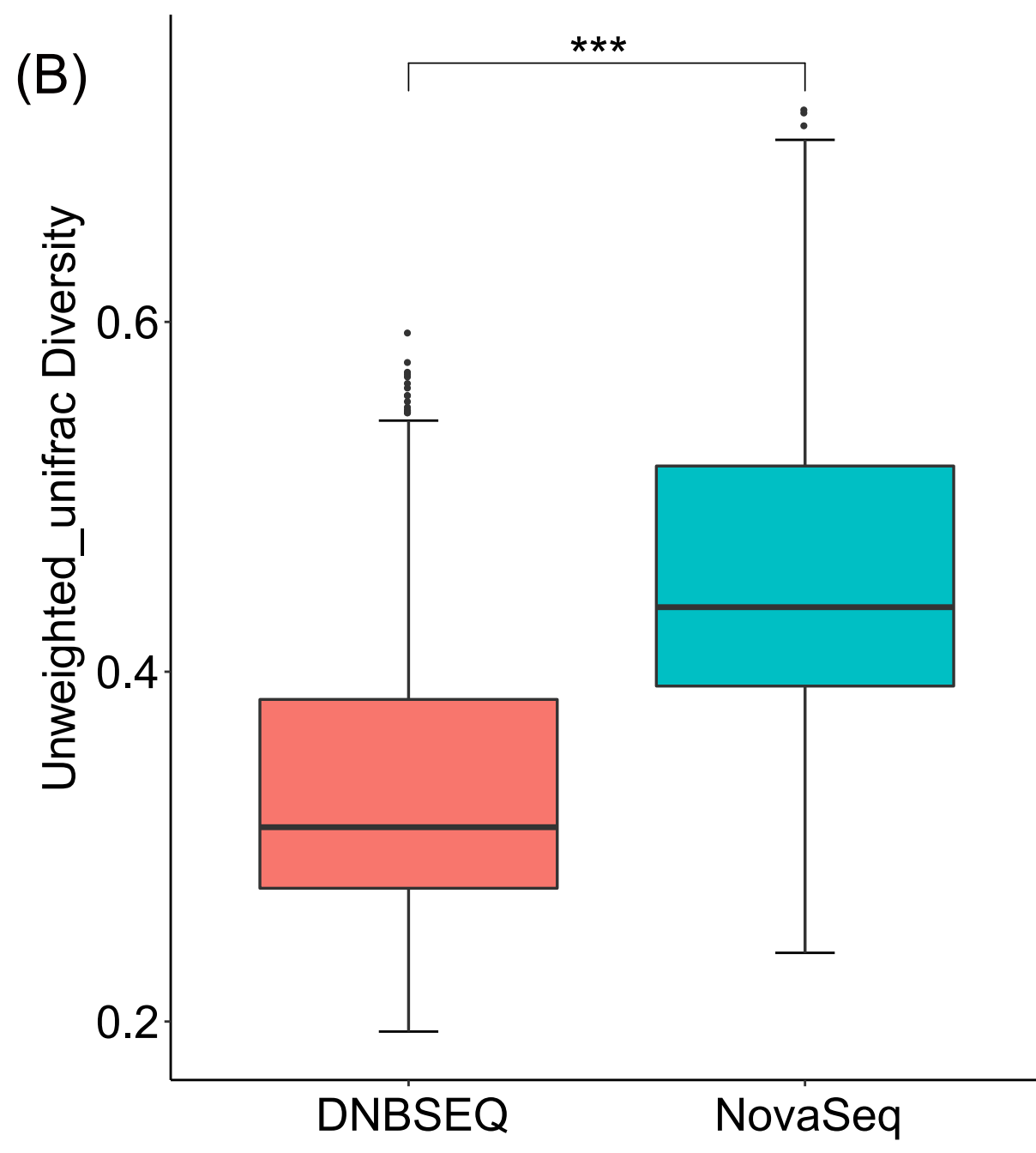

Supplement: Supplementary file 7 — Additional file 7: Figure S6. Weighted (A) and unweighted (B) UniFrac distance-based beta diversity of the cow rumen microbial communities revealed by DNBSEQ and NovaSeq sequencing platforms. [file 40793_2022_436_MOESM7_ESM.pdf]

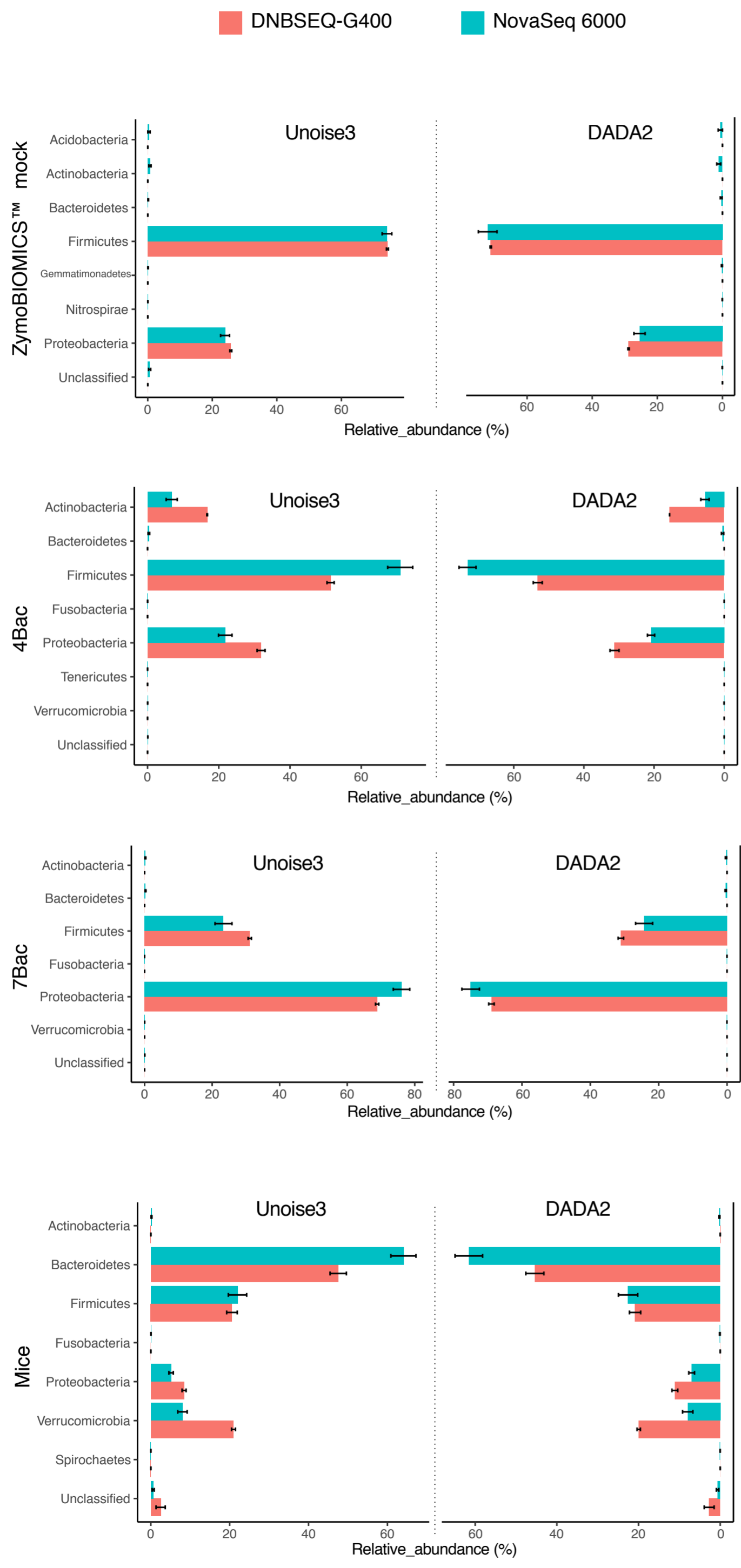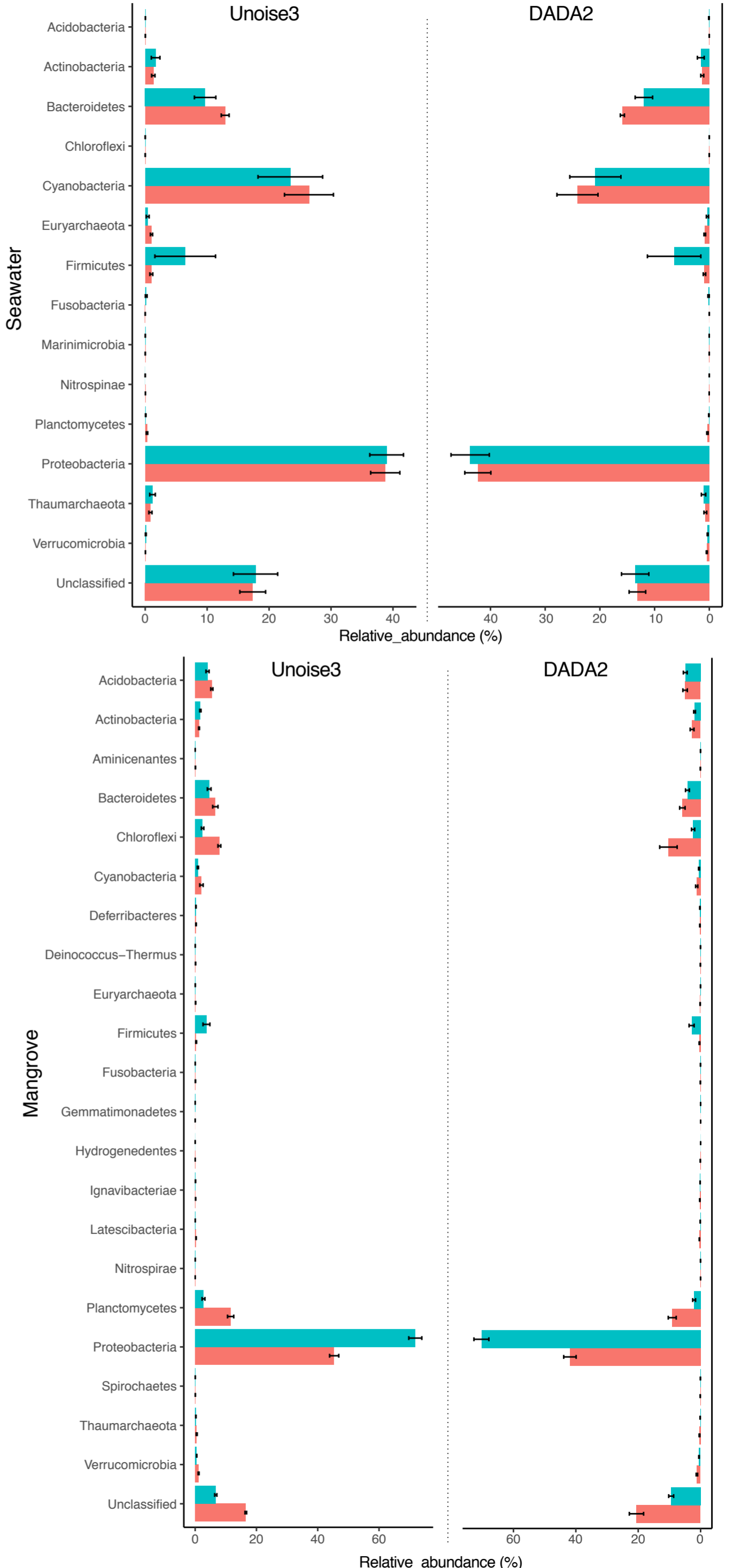

Supplement: Supplementary file 8 — Additional file 8: Figure S7. Comparison of the results using unoise3 and DADA2 denoising algorithms. The accumulated OTU relative abundances in each phylum based on unoise3 and DADA2 were graphed as bar-chart side-by-side for all the mock communities and samples from three typical ecosystems. [file 40793_2022_436_MOESM8_ESM.pdf]
