# Supplementary material for: Sequencing introduced false positive rare taxa lead to biased microbial community diversity, assembly, and interaction interpretation in amplicon studies
Source: Environ Microbiome. 2022 Aug 17;17:43. doi: 10.1186/s40793-022-00436-y (PMC9387074; doi:10.1186/s40793-022-00436-y)
Supplement: Supplementary file 6 — Additional file 6: Figure S5. Weighted (A) and unweighted (B) UniFrac distance-based clustering of the amplicon sequencing results revealed by DNBSEQ and NovaSeq sequencing platforms for samples from the three typical ecosystems. [file 40793_2022_436_MOESM6_ESM.pdf]

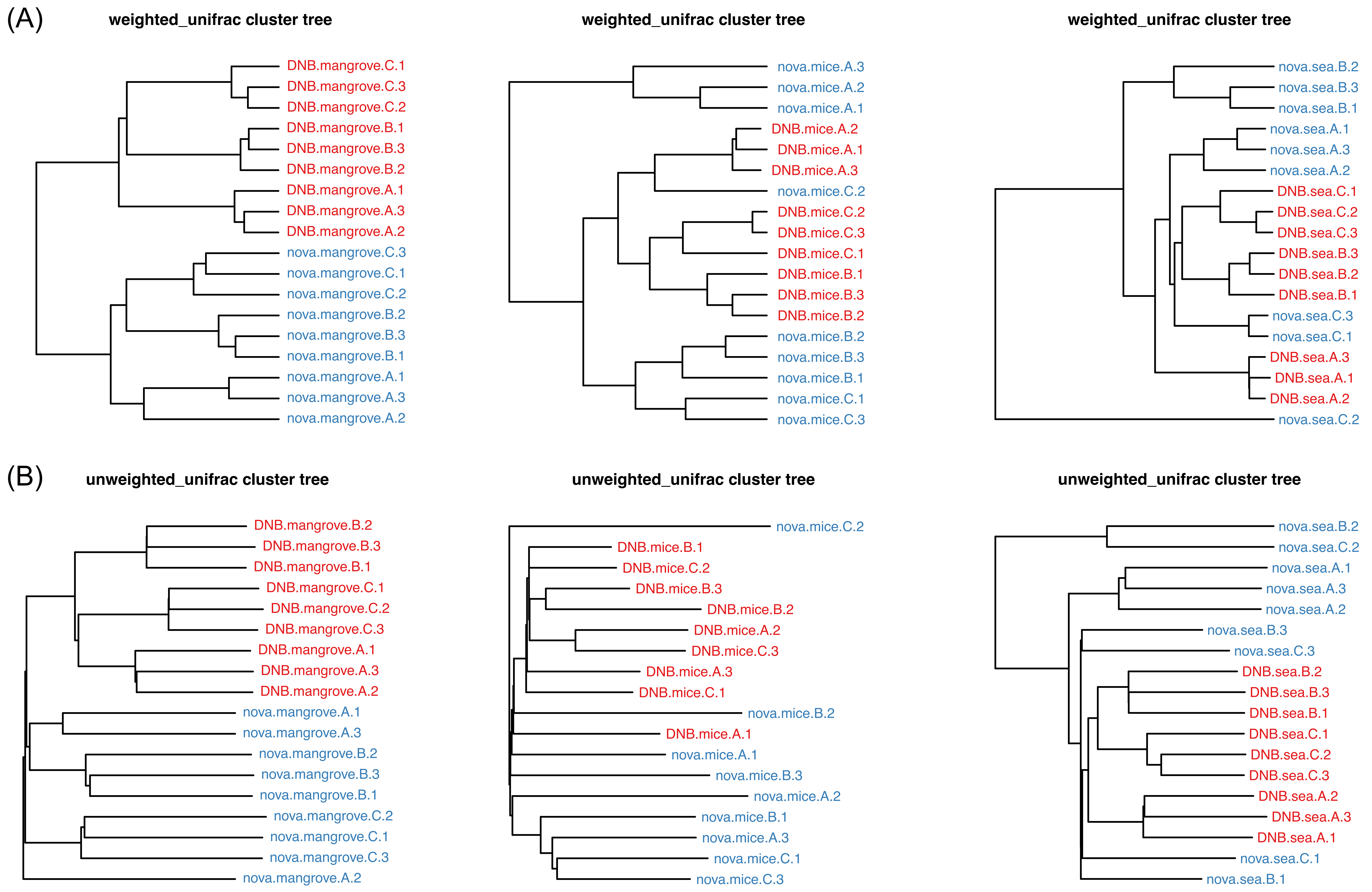
(A)
weighted_unifrac cluster tree
DNB.mangrove.C.1
DNB.mangrove.C.3
DNB.mangrove.C.2
DNB.mangrove.B.1
DNB.mangrove.B.3
DNB.mangrove.B.2
DNB.mangrove.A.1
DNB.mangrove.A.3
DNB.mangrove.A.2
nova.mangrove.C.3
nova.mangrove.C.1
nova.mangrove.C.2
nova.mangrove.B.2
nova.mangrove.B.3
nova.mangrove.B.1
nova.mangrove.A.1
nova.mangrove.A.3
nova.mangrove.A.2
weighted_unifrac cluster tree
nova.mice.A.3
nova.mice.A.2
nova.mice.A.1
DNB.mice.A.2
DNB.mice.A.1
DNB.mice.A.3
nova.mice.C.2
DNB.mice.C.2
DNB.mice.C.3
DNB.mice.C.1
DNB.mice.B.1
DNB.mice.B.3
DNB.mice.B.2
nova.mice.B.2
nova.mice.B.3
nova.mice.B.1
nova.mice.C.1
nova.mice.C.3
weighted_unifrac cluster tree
nova.sea.B.2
nova.sea.B.3
nova.sea.B.1
nova.sea.A.1
nova.sea.A.3
nova.sea.A.2
DNB.sea.C.1
DNB.sea.C.2
DNB.sea.C.3
DNB.sea.B.3
DNB.sea.B.2
DNB.sea.B.1
nova.sea.C.3
nova.sea.C.1
DNB.sea.A.3
DNB.sea.A.1
DNB.sea.A.2
nova.sea.C.2
(B)
unweighted_unifrac cluster tree
DNB.mangrove.B.2
DNB.mangrove.B.3
DNB.mangrove.B.1
DNB.mangrove.C.1
DNB.mangrove.C.2
DNB.mangrove.C.3
DNB.mangrove.A.1
DNB.mangrove.A.3
DNB.mangrove.A.2
nova.mangrove.A.1
nova.mangrove.A.3
nova.mangrove.B.2
nova.mangrove.B.3
nova.mangrove.B.1
nova.mangrove.C.2
nova.mangrove.C.1
nova.mangrove.C.3
nova.mangrove.A.2
unweighted_unifrac cluster tree
nova.mice.C.2
DNB.mice.B.1
DNB.mice.C.2
DNB.mice.B.3
DNB.mice.B.2
DNB.mice.A.2
DNB.mice.C.3
DNB.mice.A.3
DNB.mice.C.1
nova.mice.B.2
DNB.mice.A.1
nova.mice.A.1
nova.mice.B.3
nova.mice.A.2
nova.mice.B.1
nova.mice.A.3
nova.mice.C.1
nova.mice.C.3
unweighted_unifrac cluster tree
nova.sea.B.2
nova.sea.C.2
nova.sea.A.1
nova.sea.A.3
nova.sea.A.2
nova.sea.B.3
nova.sea.C.3
DNB.sea.B.2
DNB.sea.B.3
DNB.sea.B.1
DNB.sea.C.1
DNB.sea.C.2
DNB.sea.C.3
DNB.sea.A.2
DNB.sea.A.3
DNB.sea.A.1
nova.sea.C.1
nova.sea.B.1
